# Multimetallic and Mixed Environment Iridium(III) Complexes: A Modular Approach to Luminescence Tuning Using a Host Platform

**DOI:** 10.1002/chem.201700237

**Published:** 2017-05-03

**Authors:** Victoria E. Pritchard, Diego Rota Martir, Eli Zysman‐Colman, Michaele J. Hardie

**Affiliations:** ^1^ School of ChemistryUniversity of LeedsLeedsLS2 9JTUK; ^2^ Organic Semiconductor CentreEaStCHEM School of ChemistryUniversity of St. AndrewsSt Andrews, FifeKY16 9STUK

**Keywords:** cyclometallated, cyclotriveratrylene, iridium, luminescence, trinuclear complexes

## Abstract

Mononuclear and trinuclear bis‐cyclometallated Ir^III^ complexes of the host ligands tris(4‐[4′‐methyl‐2,2′‐bipyridyl]methyl)cyclotriguaiacylene (**L1**) and tris(4‐(4′‐methyl‐2,2′‐ bipyridyl)carboxy)cyclotriguaiacylene (**L2**) have been prepared. Complexes [{Ir(ppy)_2_}_3_(**L1**)](PF_6_)_3_ (**1.1**), [{Ir(ppy)_2_}(**L1**)](PF_6_)_3_ (**1.2**), [{Ir(ppy)_2_}_3_(**L2**)](PF_6_)_3_ (**2.1**) and [{Ir(ppy)_2_}(**L2**)](PF_6_)_3_ (**2.2**) (where ppy=phenylpyridinato) showed distinct photophysical properties depending on the **L** ligand. Complexes featuring the **L1** ligand were comparatively blue‐shifted in solution, with longer lifetimes and higher quantum yields. The mixed bis‐cyclometallated Ir^III^ complexes [{Ir(ppy)_2_}{Ir(dFppy)_2_}_2_(**L1**)](PF_6_)_3_ (**1.3**), [{Ir(ppy)_2_}{Ir(dFppy)_2_}_2_(**L2**)](PF_6_)_3_ (**2.3**), [{Ir(ppy)_2_}_2_{Ir(dFppy)_2_}(**L1**)](PF_6_)_3_ (**1.4**) and [{Ir(ppy)_2_}_2_{Ir(dFppy)_2_}(**L2**)](PF_6_)_3_ (**2.4**) (where dFppy=2,4‐difluorophenylpyrinato) were also synthesised. Steady‐state and time‐resolved spectroscopy, along with electrochemical investigations, show that the Ir(III) chromophores within these mixed Ir‐environment species behave as isolated centres, with no energy transfer or electronic communication between them.

## Introduction

Over the past few decades, molecular two‐ and three‐dimensional transition‐metal polynuclear complexes have been the objects of an intense research effort as photoactive materials for optoelectronic applications.^1^, ^2^, ^3^ Iridium(III) complexes display a desirable set of optoelectronic and physical properties, including colour tunability across the visible spectrum and high chemical stability, making them suitable for a wide range of applications, including solid‐state lighting,^4^ bio‐imaging^5^ and sensing.^6^ These compounds have also been exploited as building blocks for the construction of linear or branched multinuclear assemblies, with the aim to emulate the photoinduced energy and electron transfer processes exhibited by natural photosynthetic organisms.^2^, ^7^, ^8^ However, in the vast majority of these systems, each iridium centre is characterised by the same coordination environment,^9^ and surprisingly, examples of multinuclear covalently linked systems in which the Ir^III^ complexes have distinct photophysical identities are still rare.^10^

Recently we reported the preparation of two multidentate ligands tris(4‐[4′‐methyl‐2,2′‐bipyridyl]methyl)cyclotriguaiacylene, **L1**,^11^ and tris(4‐(4′‐methyl‐2,2′‐bipyridyl)carboxy)cyclotriguaiacylene, **L2**.^12^ Cyclotriguaiacylene (CTG) is part of the cyclotriveratrylene (CTV) family of host molecules; it is chiral and has a bowl‐shape.^13^ CTG‐Type ligands have been used to form mononuclear^14^ and trinuclear^15^ transition metal complexes, along with various coordination cage assemblies.^16^ Ligands **L1** and **L2** differ only in the nature of the linker group between the metal‐binding 2,2′‐bipyridine (bpy) moiety and the tribenzo[*a*,*d*,*g*]cyclononatriene core; an ether for **L1** and an ester for **L2**. By reacting these two ligands with Re(CO)_5_Br, we prepared the luminescent symmetric complexes [{Re(CO)_3_Br}_3_(**L1**)], **R1**, and [{Re(CO)_3_Br}_3_(**L2**)], **R2**.^12^ These Re^I^ complexes showed red‐shifted emissions in DMSO (*λ*_max_≈590 nm for **R1** and *λ*_max_≈650 nm for **R2**) compared to the typical emission (*λ*_max_≈585 nm) exhibited by the monomeric Re(CO)_3_Br(bpy) complex.^17^


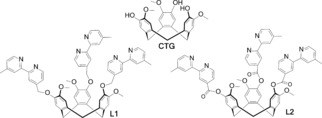


**Scheme 1:**
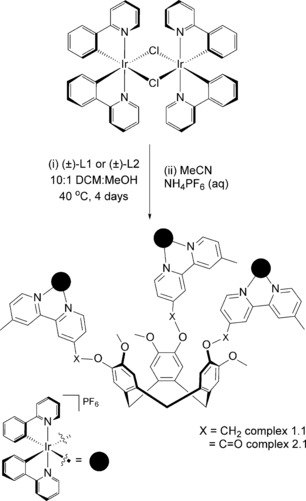
Synthesis of trinuclear symmetric complexes.

**Scheme 2:**
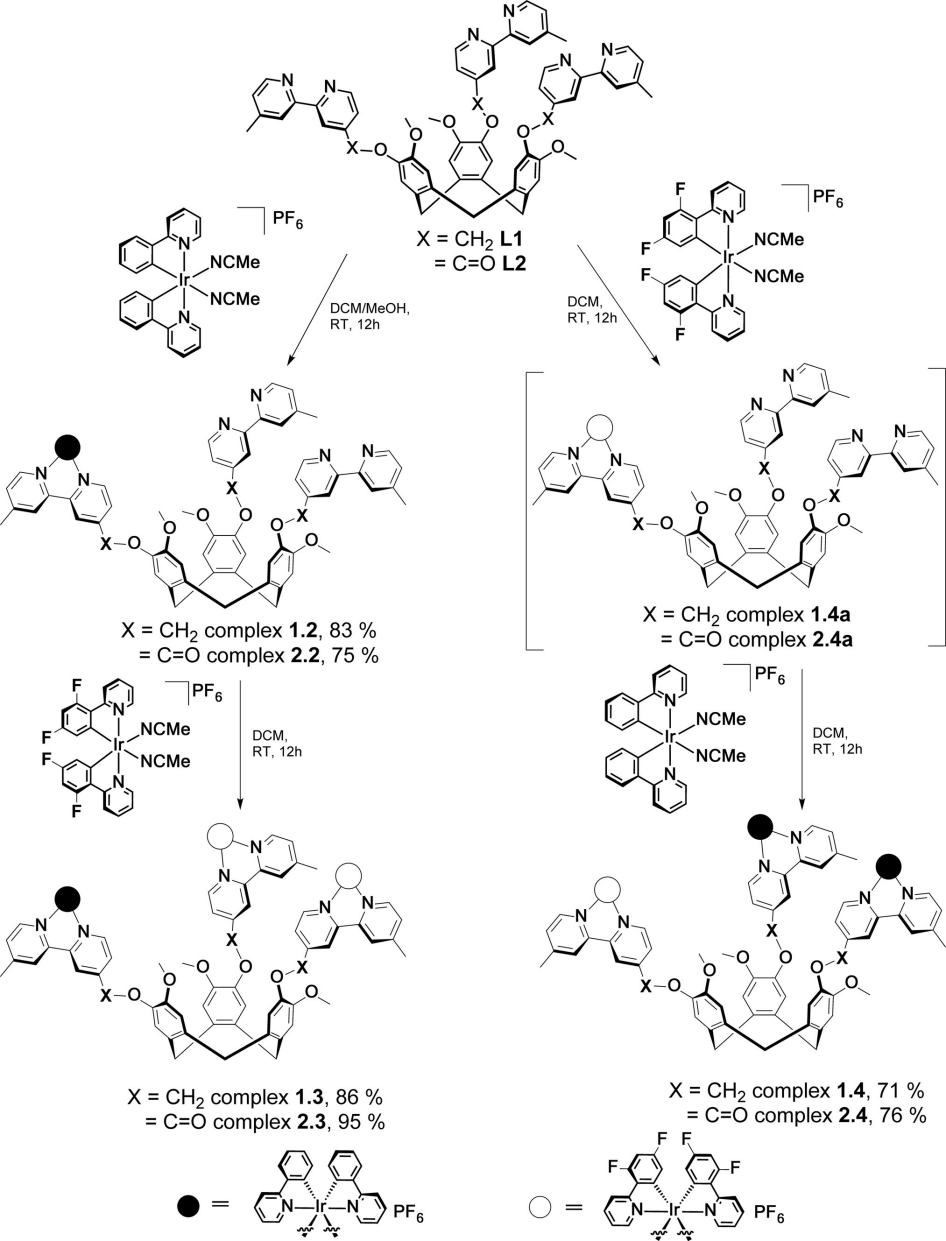
Synthesis of mononuclear and mixed trinuclear ABB‐L and BAA‐L complexes trinuclear symmetric complexes.

We report herein a series of emissive supramolecular Ir^III^ systems composed of cationic iridium complexes bearing core fragments of [Ir(ppy)_2_]^+^ and/or [Ir(dFppy)_2_]^+^ (ppy=phenylpyridinato; dFppy=2,4‐difluorophenylpyridinato) coordinated to the bpy moiety of the ancillary ligands **L1** and **L2**. We initially explored the preparation of symmetric trinuclear iridium species [{Ir(ppy)_2_}_3_(**L1**)](PF_6_)_3_ **1.1** and [{Ir(ppy)_2_}_3_(**L2**)](PF_6_)_3_ **2.1** (Scheme 1), in order to explore the influence of the linker between the CTV core and the iridium complexes (ether vs. ester, O−X in Scheme 1) on the photophysical properties of the assemblies. Only one example of a trinuclear iridium complex [{Ir(ppy)_2_}_3_(tppb)]_3_(OTf), in which tppb is the flat tripodal bridging ligand 1,3,5‐tri[3‐(2‐pyridyl)pyrazolylmethyl]‐2,4,6‐trimethylbenzene, has been previously reported.^18^ We subsequently extended our investigation to mixed multinuclear iridium systems, schematically represented as ABB‐L and BAA‐L, whereby A and B are, respectively, [Ir(ppy)_2_]^+^ and [Ir(dFppy)_2_]^+^ (complexes **1.3** and **2.3**, and **1.4** and **2.4** in Scheme 2), aiming to modulate the photophysical properties of the complexes as a function of the nature and number of the Ir^III^ species coordinated to **L1** or **L2**. The emission properties of both the trinuclear symmetric complexes (Scheme 1) and the mixed systems (Scheme 2) have been investigated in detail by steady‐state and time‐resolved spectroscopy in both solution and as polymethylmethacrylate (PMMA)‐doped films. The electrochemical properties of all the complexes have been investigated by cyclic voltammetry and differential pulse voltammetry.

## Results and Discussion

### Synthetic procedure and characterisation

**Figure 1:**
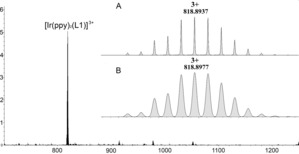
High‐Resolution ESI‐MS of complex [{Ir(ppy)_2_}_3_(**L1**)](PF_6_)_3_ **1.1** with an expanded view of the molecular peak (A) and the calculated pattern (B).

The trinuclear complexes [{Ir(ppy)_2_}_3_(**L1**)](PF_6_)_3_, **1.1**, and [{Ir(ppy)_2_}_3_(**L2**)](PF_6_)_3_, **2.1**, were obtained in good yields by reacting the (±)‐**L1** or (±)‐**L2** ligands with 1.5 equivalents of the μ‐dichloro‐bridged iridium dimer [Ir(ppy)_2_Cl]_2_ over four days, and were isolated as their PF_6_^−^ salts following an anion metathesis reaction using NH_4_PF_6_ (Scheme 1). Clear evidence of complex formation was obtained by electrospray ionisation mass spectrometry (ESI‐MS); the triply charged *m*/*z* peaks at 818.8937 for **1.1** (calculated: 818.8977, Figure 1) and 832.8730 for **2.1** (calculated: 832.870, Figure S6 in the Supporting Information) match with the expected isotope distribution patterns. Because the *M* and *P* enantiomers of the ligands (±)‐**L1** or (±)‐**L2** and the Δ and Λ enantiomers of the [Ir(ppy)_2_]^+^ moieties are present in the reaction mixtures, there are eight possible isomers for each [{Ir(ppy)_2_}_3_(**L**)]^3+^ complex, (**L**=**L1** or **L2**, Figure S1). Resolution of CTG‐type ligands is possible through chiral HPLC, however the optically active species racemise in solution on shorter timescales than those observed for the complete formation of [{Ir(ppy)_2_}_3_(**L**)]^3+^ complexes.^19^ Therefore, formation of the enantiopure [{Ir(ppy)_2_}_3_(**L**)]^3+^ complexes was not attempted.

**Figure 2:**
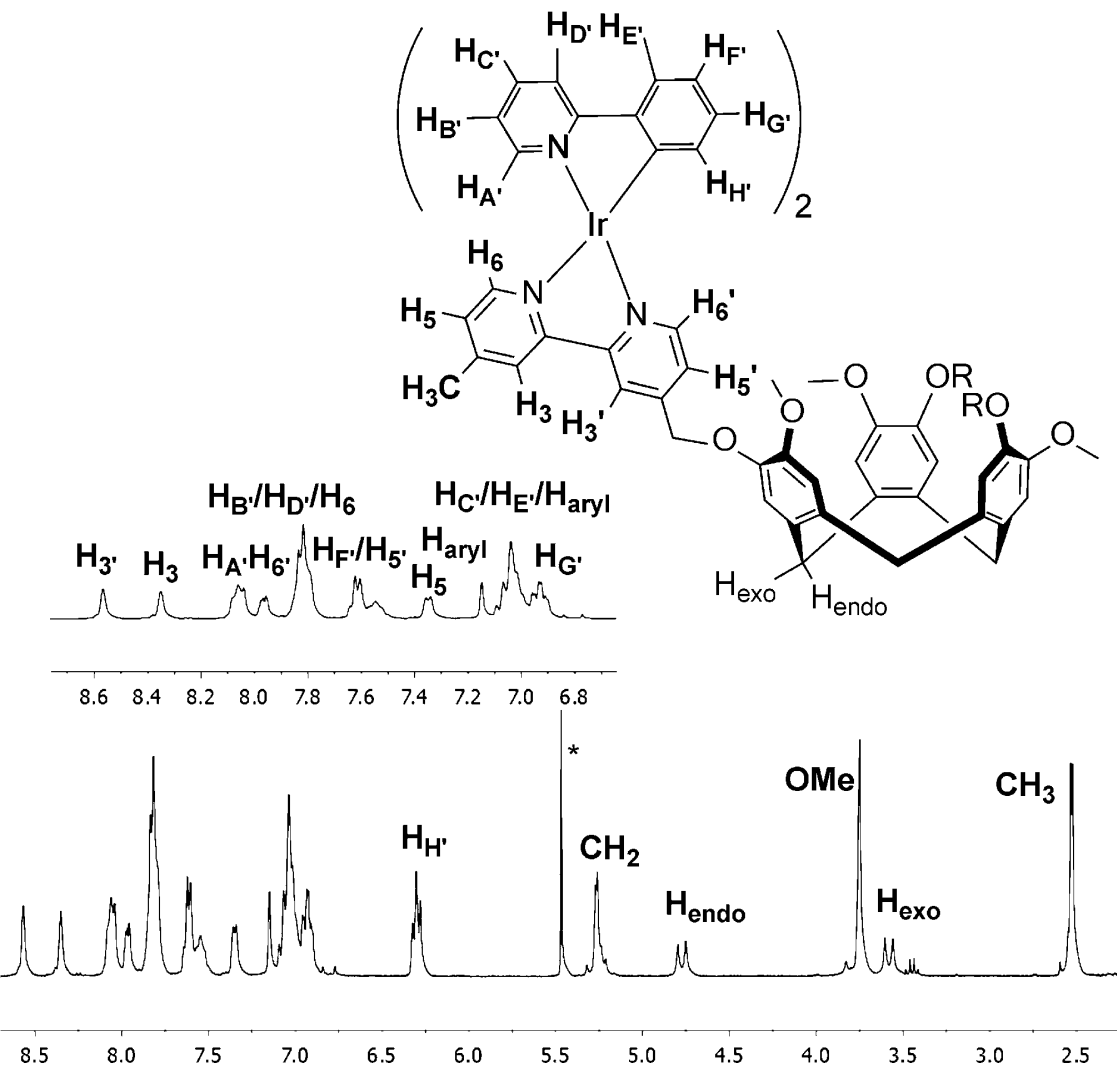
^1^H NMR (CD_3_CN) of the trinuclear complex [{Ir(ppy)_2_}_3_(**L1**)](PF_6_)_3_ **1.1** with numbering scheme and proton assignments. For clarity, only one metal‐coordinated arm of **1.1** is illustrated, trace CH_2_Cl_2_ indicated by *.

The ^1^H NMR spectra of complex **1.1** shows retention of *C*_3_‐symmetry of the **L1** ligand in CD_3_CN, peak broadening, and coordination‐induced shifts (Figure S10). The resonances of the ^1^H NMR spectrum of **1.1** (Figure 2) were assigned by using 2D ^1^H–^1^H COSY NMR experiments (Figure S11). As illustrated in Figure 2, doublets assigned to methyl, methoxy and *endo*/*exo* CH_2_ groups on ligand **L1** are all observed at the expected chemical shifts of 2.5, 3.7, 3.5 and 4.6 ppm, respectively. The OCH_2_ protons, which produce a sharp singlet in the ^1^H NMR of **L1**, are diastereomeric in the complex **1.1**, displaying a roofed doublet at 5.2 ppm. Both the resonances associated with the bpy and ppy moieties experience significant changes in chemical shift. For example, the protons located *ortho* to the *N*‐donor atoms of **L1** (H6/H6′) are shifted from approximately 8.55–8.65 ppm to 7.90–7.80 ppm after complexation. The most upshifted resonance observed at 6.20 ppm is assigned to the H_H′_ proton located on the ppy ligand. Similar spectra are observed for complex **2.1** (see Figures S12 and S13).

We also attempted to form mono‐nuclear Ir^III^ complexes by reacting only half an equivalent of [Ir(ppy)_2_Cl]_2_ with **L1** and **L2**. However, the formation of the di‐ and trinuclear species was always observed, even under careful stoichiometric control and highly diluted conditions with dropwise addition of [Ir(ppy)_2_Cl]_2_. Fortunately, by reacting one equivalent of the solvento complex [Ir(ppy)_2_(NCMe)_2_](PF_6_) with **L1** or **L2** in dichloromethane under highly diluted conditions, we successfully obtained the desired complexes [{Ir(ppy)_2_}(**L1**)](PF_6_), **1.2**, and [{Ir(ppy)_2_}(**L2**)](PF_6_), **2.2**, Scheme 2. High‐resolution ESI‐MS (Figures S3 and S7) showed the three charge states corresponding to [{Ir(ppy)_2_}(**L**)]^3+^, [{Ir(ppy)_2_}(**L**)⋅H]^2+^ and [{Ir(ppy)_2_}(**L**)⋅2 H]^+^ for each mononuclear complex. This speciation occurs because the basic nitrogen donors located on the vacant bpy moieties of **1.2** and **2.2** promote further protonation of the complexes in the gas phase. The trinuclear complexes **1.1** and **2.1** could also be prepared by reacting three equivalents of [Ir(ppy)_2_(NCMe)_2_](PF_6_) with **L1** and **L2** without any post‐synthetic salt metathesis reaction.

The ^1^H NMR spectra of the asymmetric mononuclear complexes **1.2** and **2.2** (Figures S10 and S12) are more complicated than those of the trinuclear analogues **1.1** and **2.1** due to the lower symmetry. In both cases, the spectra resemble a superposition of the ^1^H NMR spectra of the **L**‐ligand with that of the corresponding [{Ir(ppy)_2_}_3_(**L**)](PF_6_)_3_ complex. CTV‐Type ligands have been previously used to assemble trinuclear transition metal coordination complexes.^12^, ^15^ Although there have been prior examples of mononuclear complexes of CTV‐type ligands, in all cases these feature all three ligand groups that are attached to the tribenzo[*a*,*d*,*g*]cyclononatriene core binding to one metal cation,^14^ rather than having one binding ligand group and two pendant ligand groups as herein reported.

The two vacant bipyridine binding sites present in complexes **1.2** and **2.2** can be exploited for subsequent metallation steps. Addition of two equivalents of [Ir(dFppy)_2_(NCMe)_2_](PF_6_) to **1.2** and **2.2** gave rise to the formation of [{Ir(ppy)_2_}{Ir(dFppy)_2_}_2_(**L1**)](PF_6_)_3_, **1.3**, and [{Ir(ppy)_2_}{Ir(dFppy)_2_}_2_(**L2**)](PF_6_)_3_, **2.3** (Scheme 2). Conversely, when the initial formation of the monometallated species was performed with the fluorinated [Ir(dFppy)_2_(NCMe)_2_](PF_6_) complex, and the resultant mononuclear intermediates (complexes **1.4 a** and **2.4 a** in Scheme 2) were further coordinated with two equivalents of [Ir(ppy)_2_(NCMe)_2_](PF_6_), complexes [{Ir(ppy)_2_}_2_{Ir(dFppy)_2_}(**L1**)](PF_6_)_3_, **1.4**, and [{Ir(ppy)_2_}_2_{Ir(dFppy)_2_}(**L2**)](PF_6_)_3_, **2.4**, were obtained (Scheme 2). We also attempted to synthesise the pure mononuclear [{Ir(dFppy)_2_}(**L**)](PF_6_) species, but unfortunately over‐metallation and/or product degradation upon work‐up was always observed, but this can be circumvented through an in situ synthesis.

**Figure 3:**
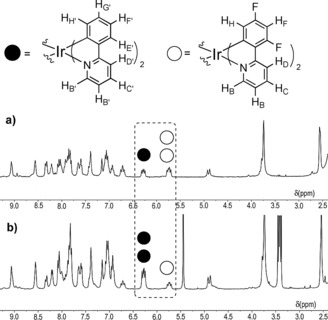
Stacked ^1^H NMR spectra (CD_3_CN) of a) **2.3** (ABB‐L type) and b) **2.4** (BAA‐L type) in CD_3_CN, displaying almost identical peak positions, but differing peak integrals commensurate with composition.

The formation of both the mononuclear [{Ir(dFppy)_2_}(**L1**)](PF_6_) **1.4 a** and [{Ir(dFppy)_2_}(**L2**)](PF_6_) **2.4 a** complexes and the mixed iridium species **1.3**, **2.3**, **1.4** and **2.4** was unequivocally confirmed in each case by ESI‐MS spectroscopy (see Figures S4, S5, S8 and S9). The ^1^H NMR spectra of complexes **2.3** and **2.4** illustrated in Figure 3 are generally broad, but show the expected features and stoichiometry. For example, in the ^1^H NMR spectrum of **2.3** (ABB‐L type), the proton resonance assigned to H_H′_ of the [Ir(ppy)_2_]^+^ moiety located at 6.2 ppm integrates to half of the resonance assigned to H_H_ of the fluorinated [Ir(dFppy)_2_]^+^ analogue located at 5.7 ppm (Figure 3 a). By contrast, in the ^1^H NMR spectrum of **2.4** (BAA‐L type, Figure 3 b), the resonance assigned to H_H′_ of [Ir(ppy)_2_]^+^ integrates to roughly twice that of the H_H_ resonance of the fluorinated [Ir(dFppy)_2_]^+^ complex. Similar features can be observed in the ^1^H NMR spectra of complexes **1.3** and **1.4** (Figure S15, S16).

### Optoelectronic properties

**Figure 4:**
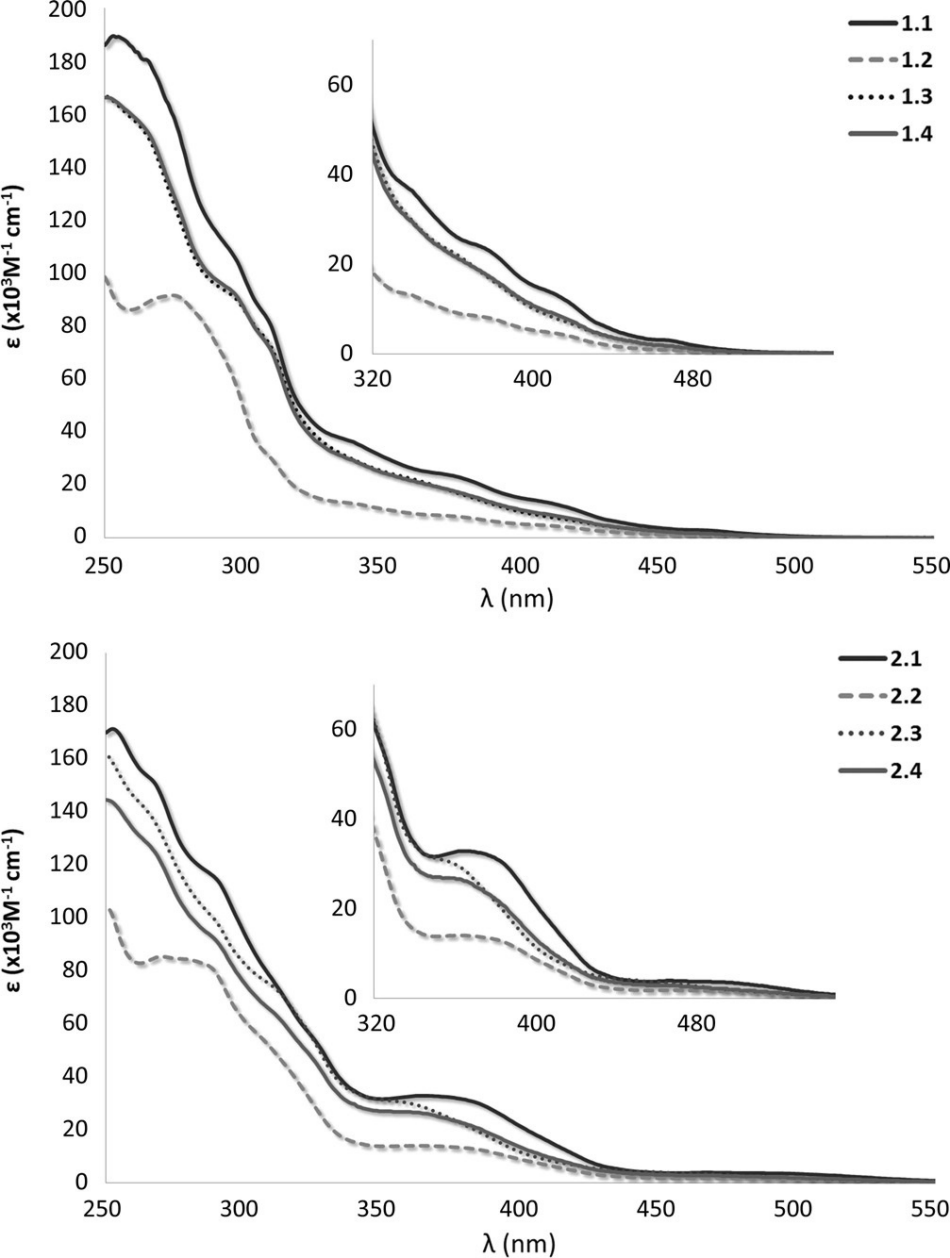
UV‐visible spectra of top) **1.1**, solid black line; **1.2**, dashed grey line; **1.3**, dotted grey line and **1.4**, solid grey line; and bottom) **2.1**, solid black line; **2.2**, dashed grey line; **2.3**, dotted grey line and **2.4**, solid grey line. The spectra were collected in CH_3_CN at 298 K.

**Figure 5:**
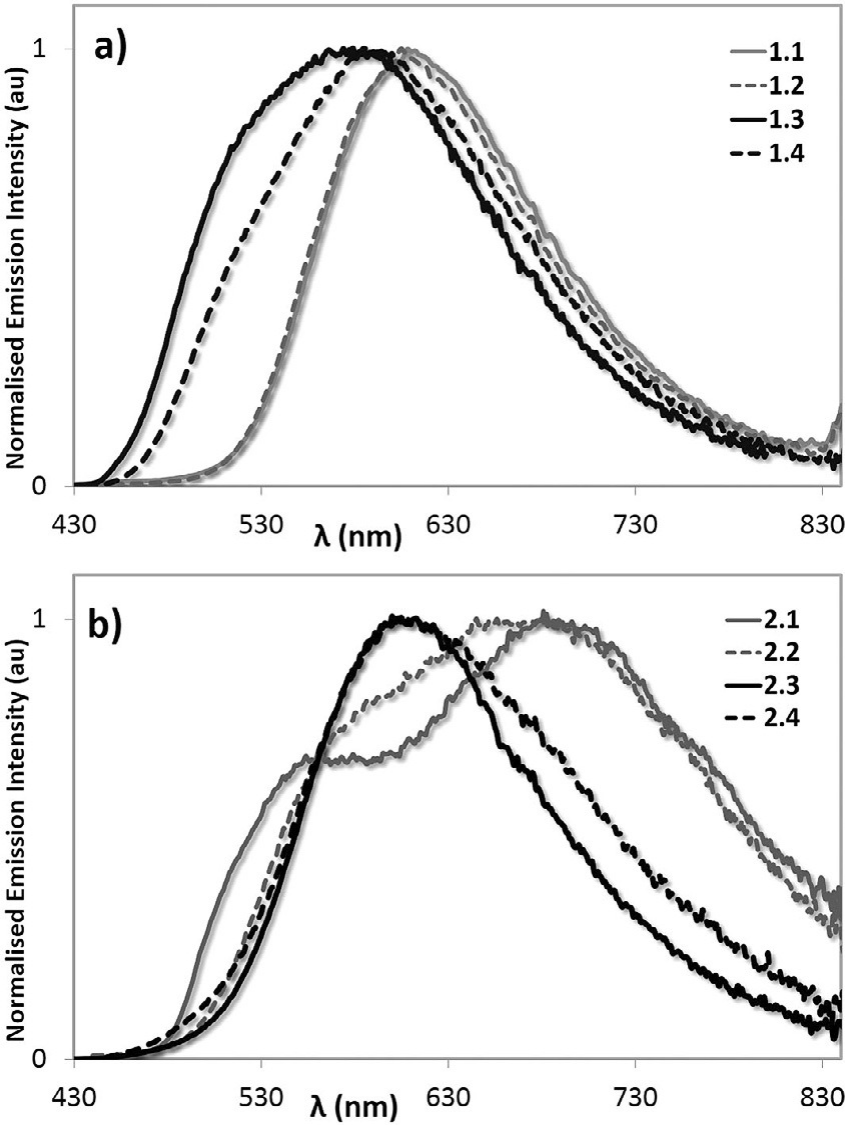
Normalised photoluminescence spectra of a) **1.1**, solid grey line; **1.2**, dashed grey line; **1.3**, solid black line and **1.4**, dashed black line; and b) **2.1**, solid grey line; **2.2**, dashed grey line; **2.3**, solid black line and **2.4**, dashed black line. The spectra were collected in degassed CH_3_CN at 298 K upon photoexcitation at 360 nm.

The optoelectronic properties of all the complexes were investigated both in CH_3_CN solution and polymethylmethacrylate (PMMA)‐doped films; these are summarised in Tables 1 and S1. The absorption spectra of both families of complexes **1.1**, **1.2**, **1.3**, **1.4** (Figure 4 a) and **2.1**, **2.2**, **2.3** and **2.4** (Figure 4 b) are all characterised by two intense bands between 260 nm and 320 nm, and broad lower‐intensity bands between 360 nm and 420 nm. Absorption spectra of the ligands are given in Figure S27. Similar to many other mononuclear iridium complexes of the structure [Ir(C^N)_2_(N^N)]^+^ reported in the literature,^20^ the higher energy bands are assigned as spin‐allowed ^1^π→π* ligand‐centred (^1^LC) transitions localised on the C^N ligand, whereas the broad bands at wavelengths longer than 340 nm are assigned as a mixture of spin‐allowed and spin‐forbidden metal‐to‐ligand and ligand‐to‐ligand charge transfer transitions (^1^MLCT/^1^LLCT and ^3^MLCT/^3^LLCT). The presence of the electron‐withdrawing ester linker between the metal‐binding bpy moiety and the CTG core in complexes **2.1**, **2.2**, **2.3**, and **2.4** produces enhanced molar absorptivities for the CT transitions occurring between 350 nm and 420 nm.^21^ In addition, due to the presence of the electron‐withdrawing fluorine atoms on the [Ir(dFppy)]^+^ scaffold, the CT transitions of **1.3** and **1.4** (*λ*_abs_ at ca. 365 nm) and **2.3** and **2.4** (*λ*_abs_ at ca. 380 nm) are slightly blue‐shifted compared to those of **1.1** and **1.2** (*λ*_abs_ at ca. 380 nm) and **2.1** and **2.2** (*λ*_abs_ at ca. 395 nm, Figure 4). Figure 5 illustrates the normalised room‐temperature emission spectra of the complexes in deuterated CH_3_CN upon excitation at 360 nm. Complexes **1.1** and **1.2** exhibit broad and unstructured yellow–orange emissions (*λ*_max_=610 nm for **1.1** and *λ*_max_=608 nm for **1.2**), with photoluminescence quantum yields (*Φ*_PL_) of 9.8 % and 14.4 %, and mono‐exponential emission lifetimes (*τ*_e_) of 294 ns and 319 ns, respectively.^22^ Compared to **1.1** and **1.2**, the mononuclear [Ir(ppy)_2_(dmbpy)](PF_6_) complex (dmbpy=4,4′‐dimethyl‐2,2′‐bipyridine) shows a blue‐shifted emission at *λ*_max_=580 nm, with an enhanced *Φ*_PL_ of 23 % and a *τ*_e_ of 310 ns.^23^ Due to the increased conjugation into the CTV scaffold promoted by the ester linker, complexes **2.1** and **2.2** exhibit broad and redshifted emissions with two maxima at 560 nm and 690 nm for **2.1** (*Φ*_PL_=1.4 %, *τ*_e_=14, 193 ns), and 565 nm and 680 nm for **2.2** (*Φ*_PL_=1.0 %, *τ*_e_=22, 398 ns). A similar emission profile featuring two emission maxima at 530 nm and 650 nm was also observed in DMSO for the previously reported [(Re(CO)_3_Br)_3_(**L2**)] complex.^12^ In addition, the photophysical properties of **2.1** and **2.2** are somewhat comparable to those of the mononuclear [Ir(ppy)_2_(mdcbpy)]PF_6_ complex (mdcbpy=dimethyl‐2,2′‐bipyridine‐4,4′‐dicarboxylate), which exhibits broad and weak emissions at 608 and 651 nm with a *Φ*_PL_ of 1.0 % in degassed CH_2_Cl_2_.^24^ It is worth noting that ligand **L2** is not emissive in degassed CH_3_CN. Therefore, we can exclude any contributions from **L2** to the broad emission observed from complexes **2.2** and **2.1**.

**Table 1:** Photophysical properties of complexes.

	*λ*_em_ [nm]^[b]^		*Φ*_PL_ [%]^[c]^		*τ*_e_ [ns]	
	CH_3_CN^[a]^	Film^[d]^	CH_3_CN^[a]^	Film^[d,e]^	CH_3_CN^[a,f]^	Film^[d,f]^
1.1	610	565	9.8	17.7	294	23 (5), 194 (35), 960 (60)
1.2	608	566	14.4	26.4	319	38 (3), 376 (26), 1210 (71)
2.1	563 (0.8), 686 (1)	625	1.4	13.9	14 (7), 193 (93)	20 (8), 248 (27), 1003 (65)
2.2	566 (0.7), 686 (1)	601	1.0	21.3	22 (4), 398 (96)	19 (12) 291 (19), 1032 (69)
1.3	574	554	5.5	23.2	267 (32), 1252 (68)	41 (4), 400 (28), 1254 (68)
1.4	596	563	4.3	17.6	285 (70), 1090 (30)	28 (5), 381 (26), 1143 (69)
2.3	608	594	2.6	21.6	185 (60), 596 (40)	23 (6), 305 (41), 958 (63)
2.4	611	615	2.0	15.7	60 (58), 233 (42)	19 (7), 252 (42), 989 (59)

[a] Measurements in degassed CH_3_CN at 298 K. [b] Principal emission peaks listed with values in parentheses indicating relative intensity. [c] Quinine sulphate employed as the external reference (*Φ*_PL_=54.6 % in 0.5 m H_2_SO_4_ at 298 K).^26^ [d] PMMA doped films (5 wt % of complex) formed by spin‐coating deposition on quartz substrate. *Φ*_PL_ measurements were carried out under N_2_. [e] Values obtained using an integrating sphere. [f] Values in parentheses are pre‐exponential weighting factor, in relative percentage intensity, of the emission decay kinetics (*λ*_exc_=378 nm).

The introduction of the [Ir(dFppy)]^+^ scaffold in the mixed multimetallic complexes [{Ir(ppy)_2_}{Ir(dFppy)_2_}_2_(**L**)](PF_6_)_3_ (**1.3** and **2.3**), and [{Ir(ppy)_2_}_2_{Ir(dFppy)_2_}(**L**)](PF_6_)_3_ (**1.4** and **2.4**), promoted the expected blue‐shifted emissions compared to the corresponding homonuclear complexes [{Ir(ppy)_2_}_3_(**L**)](PF_6_)_3_, **1.1** and **2.1** and [(Ir(ppy)_2_)(**L**)](PF_6_), **1.2** and **2.2**. The emission of complex **1.3** is blue‐shifted at *λ*_max_=580 nm with a *Φ*_PL_ of 5.5 % compared to **1.4**, which showed an unstructured emission profile with a *λ*_max_=595 nm and a *Φ*_PL_ of 4.3 %. By contrast, similar emission profiles at *λ*_max_=610 nm are observed for both the mixed‐metal complexes **2.3** (*Φ*_PL_=2.6 %) and **2.4** (*Φ*_PL_=2.0 %), which are notably sharper and more blue‐shifted compared to the homonuclear complexes **2.1** and **2.2** (Figure 5 b).

Upon photoexcitation at 378 nm, each of the complexes **1.3** and **1.4** and **2.3** and **2.4** exhibited biexponential emission decays when monitoring at their respective emission maxima. Complexes **1.3** and **1.4** exhibited *τ*_e_ values of 267, 1252 ns and 285, 1090 ns, respectively, whereas complexes **2.3** and **2.4** showed shorter biexponential decays of 185, 596 ns and 60, 233 ns, respectively. It is worth noting that, in degassed CH_3_CN, the short components of the decays of **1.3** and **1.4** (267 ns and 285 ns, respectively) are comparable to the emission decay of the mononuclear non‐fluorinated complex [Ir(ppy)_2_(dmbpy)](PF_6_) (310 ns). The short components of **2.3** and **2.4** (185 ns and 60 ns, respectively) are similar to that of the mononuclear complex [Ir(ppy)_2_(dmbpy)](PF_6_) (110 ns). Similarly, the long components of **1.3** and **1.4** (1252 ns and 1090 ns, respectively) and **2.3** and **2.4** (596 ns and 233 ns, respectively) are somewhat comparable to those of the corresponding mononuclear fluorinated complexes [Ir(dFppy)_2_(dmbpy)](PF_6_) (660 ns) and [Ir(dFppy)_2_(mdcbpy)](PF_6_) (390 ns).^21^, ^23^ In addition, the short components of the decays of **1.3** and **1.4** are in line with those of the homonuclear iridium complexes **1.1** (294 ns) and **1.2** (319 ns), whereas the short components of **2.3** and **2.4** are similar to the biexponential decays of **2.1** (14, 193 ns) and **2.2** (22, 398 ns). Considering the comparison with the emission lifetimes from these reference complexes, the biexponential decays observed for **1.3**, **1.4**, **2.3** and **2.4** are interpreted as the result of the radiative relaxation of both the nonfluorinated [Ir(ppy)_2_]^+^ complex (short components) and the fluorinated [Ir(dFppy)_2_]^+^ complex (long components). As reported in Table 1, the pre‐exponential weighting factors of the emission decay kinetics of **1.3**, **1.4**, **2.3** and **2.4** monitored at the emission maximum support the stoichiometry of the mixed multimetallic systems. Indeed, for complexes **1.3** and **2.3**, which are composed of two [Ir(dFppy)_2_]^+^ units and one [Ir(ppy)_2_]^+^ scaffold, the relative weighting of the long components is approximately twice as large as that of the short components. The opposite trend is observed for the complexes **1.4** and **2.4**.^25^ These lifetime data suggest that upon photoexcitation into the coincident CT absorption bands of both the nonfluorinated and the fluorinated iridium complexes, emission results from both chromophoric units without any electronic internuclear communication. Therefore, for **1.3**, **1.4**, **2.3** and **2.4**, it appears unlikely that there is any energy or electron transfer between the two electronically distinct iridium complexes.

To mitigate the non‐radiative vibration motion of the complexes, we spin‐coated 5 wt % thin films of **1.1**, **1.2**, **1.3** and **1.4** and **2.1**, **2.2**, **2.3** and **2.4** in PMMA, which serves as an inert matrix. For all the complexes, the emissions in thin films were blue‐shifted and sharper compared to the corresponding emissions in CH_3_CN (Figure S29). In addition, the *Φ*_PL_ of all the species were enhanced as a result of the rigidity induced by the PMMA host, and the biexponential emission lifetimes were significantly longer (see Table 1). As expected, the emission profiles of **2.1**, **2.2**, **2.3** and **2.4** (*λ*_max_=625 nm for **2.1**, *λ*_max_=601 nm for **2.2**, *λ*_max_=594 nm for **2.3** and *λ*_max_=615 nm for **2.4**) were redshifted compared to those of complexes **1.1**, **1.2**, **1.3** and **1.4** (*λ*_max_=665 nm, *λ*_max_=566 nm, *λ*_max_=554 nm and *λ*_max_=563 nm).

### Electrochemical properties

**Figure 6:**
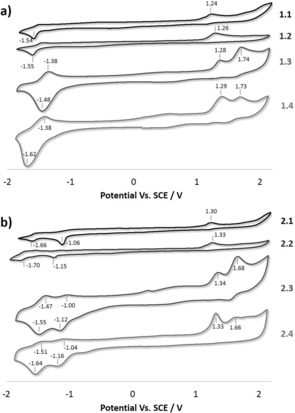
Cyclic voltammograms (CV) of a) from top to bottom: **1.1**, **1.2**, **1.3**, and **1.4**; b) from top to bottom: **2.1**, **2.2**, **2.3** and **2.4**;. The spectra were recorded at 298 K in degassed CH_3_CN solution containing *n*‐NBu_4_PF_6_ as the supporting electrolyte and using Fc/Fc^+^ as an internal standard (Fc/Fc^+^=0.38 V in CH_3_CN with respect to SCE).^29^ The CV of the mononuclear complexes **1.2** and **2.2** were collected at a concentration of approximately 3 mm, whereas the CV of the trinuclear species **1.1**, **1.3**, **1.4**, **2.1**, **2.3** and **2.4** were collected at a concentration of approximately 1 mm.

The ground state electrochemical properties of complexes **1.1**, **1.2**, **1.3**, **1.4**, **2.1**, **2.2**, **2.3** and **2.4** were investigated by cyclic voltammetry (CV) in degassed CH_3_CN (Figure 6 and Table S2). Similar to the redox properties reported for the mononuclear [Ir(ppy)_2_(dmbpy)](PF_6_) complex,^23^ both complexes **1.1** and **1.2** exhibit a single irreversible oxidation, respectively at *E* ^pa^=1.24 V and 1.26 V, respectively, assigned to the Ir(III/IV) redox couple with significant contribution from the ppy ligands, and a single irreversible reduction located at *E* ^pc^=−1.54 V for **1.1** and −1.55 V for **1.2**, which occurs on the ancillary bipyridine ligands^27^ (Figure 6 a). Although no redox processes are observed within the solvent window in the CV of **L1**, ligand **L2** exhibited a reversible one‐electron reduction at Ered1/2 =1.08 V, which is ascribed to the formation of a carboxylate radical anion (Figure S46).^28^ This reduction process is also present at *E* ^pc^=−1.06 V and *E* ^pc^=−1.15 V in the CVs of **2.1** and **2.2**, respectively (Figure 6 b). The ester moiety in **2.1** and **2.2** draws electron density away from the iridium centres.^21^ Thus, compared to **1.1** and **1.2**, the oxidation process for both **2.1** and **2.2** are slightly anodically shifted at *E* ^pa^=1.30 V and *E* ^pa^=1.33 V, respectively. The bipyridine‐based reductions are shifted to lower potentials at *E* ^pc^=−1.66 V and −1.70 V, respectively, as a consequence of the more electron‐poor nature of the ancillary ligand. The redox processes of **2.1** and **2.2** are similar to those of the mononuclear [Ir(ppy)_2_(mdcbpy)](PF_6_) complex (Eox1/2 =1.33 V, Ered1/2 =−1.00 V and Ered1/2 =−1.54 V).^21^ Two oxidation processes ascribed to the formation of the Ir^III/IV^ redox couples of both the fluorinated and non‐fluorinated Ir^III^ complexes with significant contribution from the C^N ligands are observed in the CVs of all the mixed‐Ir systems **1.3**, **1.4**, **2.3** and **2.4**. The oxidation at less‐positive potentials is due to the formation of the Ir^III/IV^ redox couple involving the [Ir(ppy)_2_]^+^ scaffold (*E* ^pa^=1.28 V for **1.3**, *E* ^pa^=1.29 V for **1.4**, *E* ^pa^=1.34 V for **2.3** and *E* ^pa^=1.33 V for **2.4**), whereas the oxidation localised at more positive potentials is due to the oxidation of the [Ir(dFppy)_2_]^+^ centre (*E* ^pa^=1.74 V for **1.3**, *E* ^pa^=1.73 V for **1.4**, *E* ^pa^=1.68 V for **2.3** and *E* ^pa^=1.66 V for **2.4**). Finally, complexes **1.3** and **1.4** each exhibit one quasi‐reversible reduction, localized at Ered1/2 =−1.42 V for **1.3** and Ered1/2 =−1.50 V for **1.4**, whereas two quasi‐reversible reductions are observed for both **2.3** (Ered1/2 =−1.06 V and Ered1/2 =−1.52 V) and **2.4** (Ered1/2 =−1.09 V and Ered1/2 =−1.57 V). For all the mixed‐Ir complexes **1.3**, **1.4**, **2.3** and **2.4**, the same redox potentials are observed in the differential pulse voltammetry (DPV) spectra (Figure S47). The electrochemistry of the mixed systems **1.3**, **1.4**, **2.3** and **2.4** suggest that overall there is no ground state electronic communication between the nonfluorinated and fluorinated iridium complexes. As a result, the redox properties observed in the CVs (Figure 6) and DPVs (Figure S47) of **1.3**, **1.4**, **2.3** and **2.4** represent a superposition of the oxidation and reduction processes exhibited by the two individual Ir^III^ complexes.

## Conclusion

In conclusion, we report a series of emissive supramolecular Ir^III^ systems composed of bis‐cyclometallated iridium(III) complexes covalently linked to bipyridine‐functionalised cyclotriguaiacylene (CTG) ligands. Depending on the nature of the linkage between the CTG core and the bipyridine chelating unit (ether vs. ester), different photophysical properties were observed. In both the CH_3_CN solution and PMMA‐doped thin films, the ester linker in complexes **2.1** and **2.2** promotes a redshifted emission, lower *Φ*_PL_, and shortened lifetimes compared to the complexes bearing the ether spacer (**1.1** and **1.2**). Mononuclear {Ir(C^N)_2_}−**L** complexes can be further metallated with distinct Ir(C^∧^N)_2_ groups to give {Ir(C^N)_2_}_3_−**L** complexes characterised by a mixture of nonfluorinated [Ir(ppy)_2_]^+^ and fluorinated [Ir(dFppy)_2_]^+^ centres (complexes **1.3**, **1.4**, **2.3** and **2.4**). Despite the covalent connection between the two Ir^III^ units and their relatively close physical proximity, no electronic communication was observed either in the ground state (CV and absorption measurements) or in the excited state (steady‐state and time‐resolved emission measurements) between the nonfluorinated and fluorinated Ir^III^ species. This is unusual behaviour for multimetallic Ir^III^‐chromophoric complexes, since energy transfer between metal centres is commonly observed.^2^, ^8^, ^30^ We believe that our approach is a promising method of incorporating a variety of chromophoric units into a single robust architecture, leading to the preparation of a large variety of multi‐chromophoric and/or multinuclear complexes. In this context, preliminary experiments show that [{Ir(ppy)_2_}{Ru(bpy)_2_}_2_(**L**)]^5+^ (**L**=**L1** or **L2**) can also be prepared. The absence of electronic communication between the chromophores means that emissive properties can be tuned through a predictable addition strategy. Furthermore, this approach offers the possibility of multi‐chromophore systems, in which only one chromophore is excited at a time, and at a particular wavelength. These systems are therefore of great interest for many applications, including optoelectronics, energy conversion, and dual‐mode synergistic photoredox catalysis.

## Experimental Section

### Synthesis

Tris(4‐(4′‐methyl‐2,2′‐bipyridyl)methyl)cyclotriguaiacylene (**L1**),^11^ tris(4‐(4′‐methyl‐2,2′‐bipyridyl)carboxy)cyclotriguaiacylene (**L2**),^12^ [Ir(ppy)_2_Cl]_2_ and [Ir(dFppy)_2_Cl]_2_ 31 were synthesised according to literature methods. All other chemicals were obtained from commercial sources and were used without further purification. NMR spectra were recorded on a Bruker DPX 300 MHz NMR spectrometer or a Bruker Avance 500 MHz NMR spectrometer. Time of flight (TOF) ESI‐MS were measured on a Bruker Maxis Impact instrument in positive‐ion mode. Infrared spectra were recorded as solid phase samples on a Bruker ALPHA Platinum ATR.

*General procedure for monomeric [Ir(C^N)_2_(NCMe)_2_]⋅PF_6_*: Adapted from the literature.^32^ [Ir(C*^*N)_2_Cl]_2_ (100 mg) and AgPF_6_ in CH_3_CN (2.1 equivalents in 60 mL) were heated overnight at 60 °C with stirring and in the absence of light. The solution was filtered through Celite to remove AgCl, the filtrate was concentrated in vacuo to around 1 mL, and diethyl ether was added to precipitate the product in near‐quantitative yields.

*[(Ir^III^(2‐phenylpyridinato)_2_)_3_(tris(4‐[4′‐methyl‐2,2′‐bipyridyl]methyl)CTG)]⋅3(PF_6_^−^) (**1.1**)*: [Ir(ppy)_2_(Cl)]_2_ (0.084 g, 0.078 mmol) and (±)‐**L1** (0.050 g, 0.052 mmol) were combined in a mixture of CH_2_Cl_2_/MeOH (10:1, 9 mL total) and heated to 40 °C. The reaction was monitored by ESI‐MS, and heating was continued until the main peak was the {[Ir(ppy)_2_]_3_(**L1**)}^3+^ cationic complex. The reaction mixture was evaporated to dryness and redissolved in CH_3_CN (5 mL). Halide exchange was accomplished through addition of an aqueous solution of NH_4_PF_6_^−^. The soluble PF_6_^−^ salt did not precipitate, and the CH_3_CN was removed in vacuo, leaving an aqueous residue that was extracted with CH_2_Cl_2_, dried over MgSO_4_ and concentrated to ≈1 mL in vacuo. Diethyl ether was added to the solution to give the product as a bright yellow powder (0.130 g, 88 % yield). ^1^H NMR (300 MHz, CD_3_CN): *δ* 8.52 (s, 1 H), 8.30 (s, 1 H), 8.00 (d, *J=*6.6 Hz, 2 H), 7.91 (d, *J=*3.2 Hz, 1 H), 7.78 (d, *J=*5.5 Hz, 5 H), 7.56 (d, *J=*5.5 Hz, 2 H), 7.50 (s, 1 H), 7.30 (d, *J=*5.3 Hz, 1 H), 7.10 (s, 1 H), 6.99 (t, *J=*7.7 Hz, 5 H), 6.88 (t, *J=*7.0 Hz, 2 H), 6.25 (t, *J=*6.6 Hz, 2 H), 5.22 (d, *J=*3.1 Hz, 2 H), 4.72 (d, *J=*13.7 Hz, 1 H), 3.70 (s, 3 H), 3.53 (d, *J=*13.6 Hz, 1 H), 2.47 ppm (s, 3 H); FT‐IR: $\tilde{\nu }$ =556, 737, 756, 835, 1031, 1144, 1267, 1421, 1477, 1508, 1607 (sh), 3044 cm^−1^ (br) TOF‐MS ESI: *m*/*z=*818.9015 [*M*]^3+^; elemental analysis for C_126_H_102_F_18_Ir_3_N_12_O_6_P_3_ (%) calcd: C 52.33, H 3.56, N 5.81; found: C 52.40, H 3.60, N 5.70.

*[(Ir^III^(2‐phenylpyridinato)_2_)(tris(4‐[4′‐methyl‐2,2′‐bipyridyl]methyl)CTG)]⋅(PF_6_^−^) (**1.2**)*: [Ir(ppy)_2_(NCMe)_2_](PF_6_) (0.035 g, 0.048 mmol) in CH_2_Cl_2_ (100 mL) was added dropwise over a period of 1 h to a stirring solution of (±)‐**L1** (0.046 g, 0.048 mmol) in a mixture of CH_2_Cl_2_/MeOH (10:1, 100 mL total) at room temperature. Over time, after addition of the pale yellow iridium precursor solution to the colourless ligand solution, the reaction mixture became bright yellow and was analysed by ESI‐MS, stirring was continued until the main peak was the {[Ir(ppy)_2_](**L1**)}^+^ cationic complex. The reaction mixture was evaporated to dryness, redissolved in CH_3_CN then filtered through Celite to remove any unreacted **L1**. The CH_3_CN solution was concentrated in vacuo and diethyl ether was added to the solution to give the title product as a bright yellow powder (0.063 g, 82.8 % yield). ^1^H NMR (300 MHz, CD_2_Cl_2_): *δ* 8.69–8.55 (m, 1 H), 8.49 (dd, *J=*9.6, 5.3 Hz, 1 H), 8.32 (dd, *J=*15.3, 5.7 Hz, 1 H), 7.94 (d, *J=*7.6 Hz, 1 H), 7.83 (d, *J=*5.6 Hz, 1 H), 7.75 (dd, *J=*12.6, 7.1 Hz, 1 H), 7.51 (t, *J=*5.6 Hz, 1 H), 7.38 (d, *J=*5.7 Hz, 1 H), 7.24 (d, *J=*5.4 Hz, 1 H), 7.16 (d, *J=*6.5 Hz, 1 H), 7.12–6.91 (m, 2 H), 6.88 (d, *J=*2.7 Hz, 1 H), 6.79–6.67 (m, 1 H), 6.32 (d, *J=*7.5 Hz, 1 H), 5.15 (d, *J=*3.0 Hz, 1 H), 4.72 (dd, *J=*14.0, 8.6 Hz, 1 H), 3.91–3.74 (m, 1 H), 3.67 (dd, *J=*12.8, 2.6 Hz, 2 H), 3.50 (dd, *J=*14.0, 5.1 Hz, 1 H), 2.58 (s, 1 H), 2.44 ppm (s, 2 H); FT‐IR: $\tilde{\nu }$ =556, 737, 756, 839, 1031, 1144, 1266, 1422, 1477, 1508, 1606 (sh), 3052 cm^−1^ (br); TOF‐MS ESI: *m*/*z=*1455.5051 [*M*]^+^; elemental analysis for C_82_H_70_F_6_IrN_8_O_6_P⋅CH_2_Cl_2_ (%) calcd: C 59.14, H 4.30, N 6.65; found: C 58.65, H 4.30, N 6.70.

*[(Ir^III^(2‐(2,4‐difluorophenyl)pyridinato)_2_)_2_(Ir^III^(2‐phenylpyridinato)_2_)(tris(4‐[4′‐methyl‐2, 2′‐bipyridyl]methyl)CTG)]⋅3(PF_6_^−^) (**1.3**)*: [Ir(dFppy)_2_(CH_3_CN)_2_].PF_6_ (0.020 g, 0.025 mmol) was dissolved in CH_2_Cl_2_ (10 mL) and added to a stirred solution of 1.2 (0.020 g, 0.0125 mmol) in CH_2_Cl_2_ (10 mL) in the absence of light. The mixture was stirred at room temperature for 12 h until HR‐MS analysis showed full conversion to the {[Ir(ppy)_2_][Ir(dFppy)_2_]_2_(L1)}^3+^ species. The CH_2_Cl_2_ was removed in vacuo, and the residue redissolved in minimal CH_2_Cl_2_ then diethyl ether was added to the solution to give the title product as a bright yellow powder (0.032 g, 86.4 % yield). ^1^H NMR (300 MHz, CD_2_Cl_2_): *δ* 8.68 (d, *J=*11.2 Hz, 1 H), 8.36 (dd, *J=*27.8, 9.5 Hz, 1 H), 7.94 (d, *J=*7.1 Hz, 1 H), 7.90–7.69 (m, 1 H), 7.66 (d, *J=*5.3 Hz, 1 H), 7.62–7.37 (m, 1 H), 7.27 (dd, *J=*18.7, 4.9 Hz, 1 H), 7.01 (ddd, *J=*29.5, 13.9, 6.0 Hz, 2 H), 6.60 (t, *J=*10.5 Hz, 1 H), 6.32 (d, *J=*7.3 Hz, 1 H), 5.76 (d, *J=*8.2 Hz, 1 H), 5.33 (s, 1 H), 4.73 (d, *J=*13.8 Hz, 1 H), 3.83 (s, 1 H), 3.60 (d, *J=*13.4 Hz, 1 H), 2.60 ppm (d, *J=*7.6 Hz, 1 H); FT‐IR: $\tilde{\nu }$ =556, 737, 756, 837, 1030, 1145, 1267, 1405, 1426, 1478, 1509, 1603 (sh), 3066 cm^−1^ (br); TOF‐MS ESI: *m*/*z=*866.8737 [*M*]^3+^; elemental analysis for C_126_H_94_F_26_Ir_3_N_12_O_6_P_3_⋅CH_2_Cl_2_ (%) calcd: C 48.88, H 3.10, N 5.38; found: C 48.35, C 3.10, N 5.30.

*[(Ir^III^(2‐(2,4‐difluorophenyl)pyridinato)_2_)(Ir^III^(2‐phenylpyridinato)_2_)_2_(tris(4‐[4′‐methyl‐2, 2′‐bipyridyl]methyl)CTG)]⋅3(PF_6_^−^) (**1.4**)*: [Ir(dFppy)_2_(CH_3_CN)_2_].PF_6_ (0.036 g, 0.045 mmol) in CH_2_Cl_2_ (100 mL) was added dropwise over a period of 3 h to a stirred solution of (±)‐L1 (0.050 g, 0.052 mmol) in a mixture of CH_2_Cl_2_/MeOH (10:1) (150 mL) at room temperature. Over time, the reaction mixture became bright yellow and was followed by ESI‐MS. The solvent was removed in vacuo, and residue redissolved in CH_3_CN then filtered through Celite to remove any unreacted **L1**. The CH_3_CN was removed in vacuo and the resultant residue of complex **1.4 a** was redissolved in CH_2_Cl_2_ and employed directly in the next step (TOF‐MS ESI for complex **1.4 a**: *m*/*z=*1527.4680 [*M*]^+^). [Ir(ppy)_2_(CH_3_CN)_2_](PF_6_) (0.026 g, 0.035 mmol) was dissolved in CH_2_Cl_2_ (5 mL) and added to a stirred solution of [{Ir(dFppy)_2_}(**L1**)]⋅PF_6_ (**1.4** **a**) (0.030 g, 0.017 mmol) in CH_2_Cl_2_ (10 mL) in the absence of light. The mixture was stirred at room temperature for 12 h until ESI‐MS showed full conversion to **1.4**. The CH_2_Cl_2_ was removed in vacuo, and the residue re‐dissolved in minimal CH_2_Cl_2_ then diethyl ether was added to the solution to give the title product as a bright yellow powder (0.038 g, 71 % yield) ^1^H NMR (300 MHz, CD_3_CN): *δ* 8.55 (s, 1 H), 8.32 (d, *J=*8.0 Hz, 2 H), 8.03 (d, *J=*7.6 Hz, 1 H), 7.94 (d, *J=*5.8 Hz, 1 H), 7.91–7.71 (m, 4 H), 7.58 (dd, *J=*16.2, 11.3 Hz, 3 H), 7.35 (dd, *J=*11.4, 5.8 Hz, 1 H), 7.13 (s, 1 H), 7.03 (dd, *J=*15.6, 7.1 Hz, 4 H), 6.90 (dd, *J=*8.2, 6.5 Hz, 1 H), 6.69 (dd, *J=*20.5, 10.7 Hz, 1 H), 6.28 (t, *J=*6.4 Hz, 1 H), 5.74 (t, *J=*6.8 Hz, 1 H), 5.25 (t, *J=*4.6 Hz, 2 H), 4.75 (d, *J=*13.7 Hz, 1 H), 3.74 (d, *J=*1.4 Hz, 3 H), 3.56 (d, *J=*14.0 Hz, 1 H), 2.57–2.42 ppm (m, 3 H); FT‐IR: $\tilde{\nu }$ =556, 737, 756, 835, 1031, 1145, 1267, 1405, 1424, 1478, 1509, 1605 (sh), 3044 cm^−1^ (br); TOF‐MS ESI: *m*/*z=*842.8821 [*M*]^+^; elemental analysis for C_126_H_98_F_22_Ir_3_N_12_O_6_P_3_ (%) calcd: C 51.06, H 3.33, N 5.67; found: C 50.89, H 3.46, N 5.59.

*[(Ir^III^(2‐phenylpyridinato)_2_)_3_(tris(4‐[4′‐methyl‐2,2′‐bipyridyl]carboxy)CTG)]⋅3(PF_6_^−^) (**2.1**)*: An identical procedure to that of complex **1.1** was followed using (±)‐**L2** (0.050 g, 0.050 mmol) to give complex **2.1** as a bright orange powder (0.092 g, 62 % yield). ^1^H NMR (300 MHz, CD_3_CN): *δ* 9.06 (s, 1 H), 8.55 (s, 1 H), 8.21 (d, *J=*5.9 Hz, 1 H), 8.04 (m, 3 H), 7.83 (m, 4 H), 7.72‐7.50 (m, 2 H), 7.38 (s, 2 H), 7.26–6.80 (m, 6 H), 6.28 (dd, *J=*13.1, 6.9 Hz, 2 H), 4.90 (d, *J=*14.3 Hz, 1 H), 3.76 (d+s, *J=*12.2 Hz, 4 H), 2.54 ppm (s, 3 H); FT‐IR, 556, 738, 756, 837, 1031, 1138, 1177, 1250, 1417, 1478, 1608, 1750 (sh), 3050 cm^−1^ (br); TOF‐MS ESI: *m*/*z=*832.8768 [*M*]^3+^; elemental analysis for C_126_H_96_F_18_Ir_3_N_12_O_9_P_3_⋅2(CH_2_Cl_2_) (%) calcd: C 49.54, H 3.25, N 5.42; found: C 49.40, H 3.20, N 5.10.

*[(Ir^III^(2‐phenylpyridinato)_2_)(tris(4‐[4′‐methyl‐2,2′‐bipyridyl]carboxy)CTG)]⋅(PF_6_^−^) (**2.2**)*: An identical procedure to that of complex **1.2** was followed using (±)‐**L2** (0.050 g, 0.050 mmol) to give complex **2.2** as a bright pale orange powder (0.062 g, 75 % yield) ^1^H NMR (300 MHz, CD_2_Cl_2_): *δ* 9.02 (d, *J=*4.1 Hz, 1 H), 8.84 (d, *J=*5.6 Hz, 1 H), 8.54 (d, *J=*4.6 Hz, 1 H), 8.38 (s, 1 H), 8.30 (s, 1 H), 8.22 (d, *J=*6.7 Hz, 1 H), 8.12–7.90 (m, 2 H), 7.88 (d, *J=*6.0 Hz, 1 H), 7.77 (dd, *J=*17.2, 8.1 Hz, 2 H), 7.52 (dd, *J=*9.4, 5.8 Hz, 1 H), 7.42–7.19 (m, 2 H), 7.14–6.88 (m, 3 H), 6.32 (t, *J=*7.8 Hz, 1 H), 4.89 (d, *J=*14.8 Hz, 1 H), 3.78 (t, *J=*10.1 Hz, 4 H), 2.60 (s, 1 H), 2.48 ppm (s, 2 H); FT‐IR: $\tilde{\nu }$ =557, 755, 840, 1031, 1138, 1178, 1237, 1418, 1478, 1608, 1747 (sh), 3028 cm^−1^ (br); TOF‐MS ESI: *m*/*z=*1497.4428 [*M*]^+^; elemental analysis for C_82_H_64_F_6_IrN_8_O_9_P⋅(CH_2_Cl_2_) (%) calcd: C 57.71, H 3.85, N 6.49; found: C 57.80, H 3.70, N 6.35.

*[(Ir^III^(2‐(2,4‐difluorophenyl)pyridinato)_2_)_2_(Ir^III^(2‐phenylpyridinato)_2_)(tris(4‐[4′‐methyl‐2, 2′‐bipyridyl]carboxy)CTG)]⋅3(PF_6_^−^) (**2.3**)*: An identical procedure to that of complex **1.3** was followed using **2.2** (0.02 g, 0.012 mmol) to give complex **2.3** as a pale orange powder (0. 035 g, 94.5 % yield). ^1^H NMR (300 MHz, CD_2_Cl_2_): *δ* 9.04 (d, *J=*11.2 Hz, 1 H), 8.40 (d, *J=*11.2 Hz, 1 H), 8.32 (d, *J=*9.7 Hz, 1 H), 8.20 (t, *J=*5.9 Hz, 1 H), 8.07 (dd, *J=*18.4, 6.8 Hz, 1 H), 7.95 (d, *J=*9.0 Hz, 1 H), 7.90–7.63 (m, 1 H), 7.63–7.44 (m, 1 H), 7.42–7.18 (m, 1 H), 7.19–6.85 (m, 2 H), 6.72–6.46 (m, 1 H), 6.32 (t, *J=*7.7 Hz, 1 H), 5.75 (td, *J=*8.3, 2.2 Hz, 1 H), 4.87 (d, *J=*13.7 Hz, 1 H), 3.76 (d, *J=*12.0 Hz, 1 H), 2.61 ppm (d, *J=*7.1 Hz, 1 H); FT‐IR: $\tilde{\nu }$ =556, 755, 836, 1031, 1139, 1166, 1248, 1407, 1478, 1603, 1751 (sh), 3084 cm^−1^ (br); TOF‐MS ESI: *m*/*z=*880.8553 [*M*]^3+^; elemental analysis for C_126_H_89_F_25_Ir_3_N_12_O_9_P_3_ (%) calcd: C 49.46, H 2.93, N 5.48; found: C 49.09, H 3.06, N 5.39.

*[(Ir^III^(2‐(2,4‐difluorophenyl)pyridinato)_2_)(Ir^III^(2‐phenylpyridinato)_2_)_2_(tris(4‐[4′‐methyl‐2, 2′‐bipyridyl]carboxy)CTG)]⋅3(PF_6_^−^) (**2.4**)*: An identical procedure to that of complex **1.4** was followed using (±)‐**L2** (0.050 g) in the initial step and [{Ir(dFppy)_2_}(L2)]⋅PF_6_ **2.4 a** (0.015 g, 0.008 mmol) in the second step to give complex **2.4** as a pale orange powder (0.020 g, 76 % yield). ^1^H NMR (300 MHz, CD_3_CN): *δ* 9.06 (s, 1 H), 8.56 (s, 1 H), 8.33 (d, *J=*7.7 Hz, 1 H), 8.21 (d, *J=*5.0 Hz, 1 H), 8.05 (t, *J=*11.8 Hz, 2 H), 7.84 (dd, *J=*15.8, 7.1 Hz, 3 H), 7.72–7.52 (m, 1 H), 7.39 (s, 1 H), 7.15 (s, 1 H), 7.12–6.97 (m, 2 H), 6.93 (t, *J=*7.3 Hz, 1 H), 6.83–6.57 (m, 1 H), 6.28 (dd, *J=*13.0, 7.7 Hz, 1 H), 5.74 (dd, *J=*13.9, 8.6 Hz, 1 H), 4.90 (d, *J=*13.8 Hz, 1 H), 3.76 (d, *J=*13.4 Hz, 3 H), 2.54 ppm (s, 2 H); FT‐IR: $\tilde{\nu }$ =557, 755, 839, 1031, 1139, 1176, 1248, 1410, 1478, 1606, 1751 (sh), 3040 cm^−1^ (br); TOF‐MS ESI: *m*/*z=*856.8657 [*M*]^3+^; elemental analysis for C_126_H_92_F_22_Ir_3_N_12_O_9_P_3_⋅2(CH_2_Cl_2_) (%) calcd: C 48.41, H 3.05, N 5.29; found: C 47.99, H 3.26, N 5.45.

### Photophysical Measurements

All samples were prepared in HPLC grade acetonitrile with varying concentrations in the order of 10^−4^–10^−6^ m. Absorption spectra were recorded at room temperature using a Shimadzu UV‐1800 double‐beam spectrophotometer. Molar absorptivity determination was verified by linear least‐squares fit of values obtained from at least four independent solutions at varying concentrations with absorbance ranging from 6.05×10^−5^ to 2.07×10^−5^ m. PMMA‐doped films were prepared by spin‐coating the samples from a solution of 2‐methoxyethanol (HPLC grade) containing 5 % (w/w) of the desired sample. Steady‐state emission and excitation spectra and time‐resolved emission spectra of both CH_3_CN solutions and doped films were recorded at 298 K using an Edinburgh Instruments F980 device. Solid‐state PLQY measurements of thin‐films were performed in an integrating sphere under a nitrogen purge in a Hamamatsu C9920‐02 luminescence measurement system.^33^ See the Supporting Information for further details.

### Electrochemical studies

Cyclic voltammetry (CV) and Differential Pulse Voltammetry (DPV) measurements were performed on an Electrochemical Analyzer potentiostat model 600D from CH Instruments. Solutions for CV and DPV were prepared in MeCN at a concentration of approximately 3 mM for **1.2** and **2.2** and of approximately 1 mM for **1.1**, **1.3**, **1.4**, **2.1**, **2.3** and **2.4** and degassed with MeCN‐saturated nitrogen by bubbling for about 10 min prior to scanning. Tetra(n‐butyl ammoniumhexafluorophosphate (TBAPF_6_; ca. 0.1 M in MeCN) was used as the supporting electrolyte. An Ag/Ag^+^ electrode (silver wire in a solution of 0.1 M KCl in H_2_O) was used as the pseudoreference electrode; a Pt electrode was used for the working electrode and a Pt electrode was used as the counter electrode. The redox potentials are reported relative to a saturated calomel electrode (SCE) electrode with a ferrocene/ferrocenium (Fc/Fc^+^) redox couple as an internal reference (0.38 V vs. SCE).^29^

## Conflict of interest

The authors declare no conflict of interest.

## Supporting information

As a service to our authors and readers, this journal provides supporting information supplied by the authors. Such materials are peer reviewed and may be re‐organized for online delivery, but are not copy‐edited or typeset. Technical support issues arising from supporting information (other than missing files) should be addressed to the authors.

Supplementary
