# Supplementary material for: Multimetallic and Mixed Environment Iridium(III) Complexes: A Modular Approach to Luminescence Tuning Using a Host Platform
Source: Chemistry. 2017 May 3;23(37):8839–49. doi: 10.1002/chem.201700237 (PMC5499719; doi:10.1002/chem.201700237)
Supplement: Supplementary file 1 — Supplementary [file CHEM-23-8839-s001.pdf]

CHEMISTRY
A European Journal

Supporting Information

Multimetallic and Mixed Environment Iridium(III) Complexes: A Modular Approach to Luminescence Tuning Using a Host Platform

Victoria E. Pritchard+,[a] Diego Rota Martir+,[b] Eli Zysman-Colman,*[b] and Michaele J. Hardie*[a]

chem_201700237_sm_miscellaneous_information.pdf

Supporting Information

Multimetallic and mixed environment iridium(III) complexes: A modular approach to luminescence tuning using a host platform.

V. E. Pritchard,[a] D. Rota Martir,[b] E. Zysman-Colman[b] and M. J. Hardie[a]

[a] Dr V. E. Pritchard, Prof. M. J. Hardie, School of Chemistry, University of Leeds, Woodhouse Lane, Leeds LS2 9JT, UK

[b] Mr D. Rota Martir, Dr E. Zysman-Colman, Organic Semiconductor Centre, EaSTCHEM School of Chemistry, University of St Andrews, St Andrews, Fife KY16 9ST, UK

1. Additional Charts
2. Mass Spectrometry
3. NMR
4. Photophysical measurements
5. Electrochemistry

1. Additional Charts

Chart of complexes in this study:

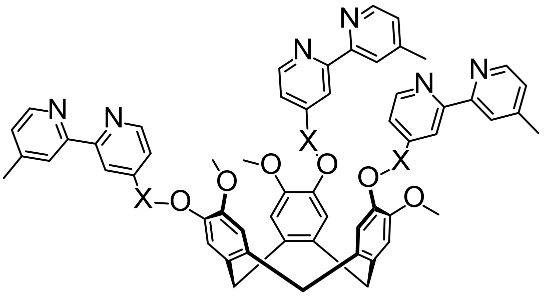

X = CH_2 ligand **L1**
= C=O ligand **L2**

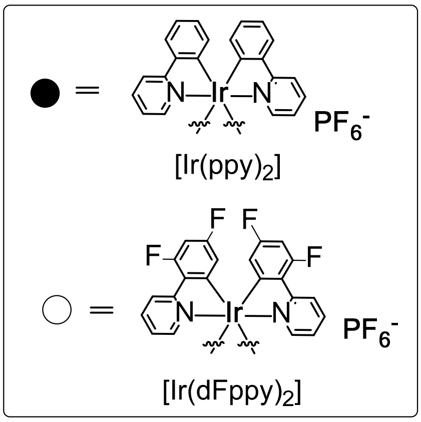

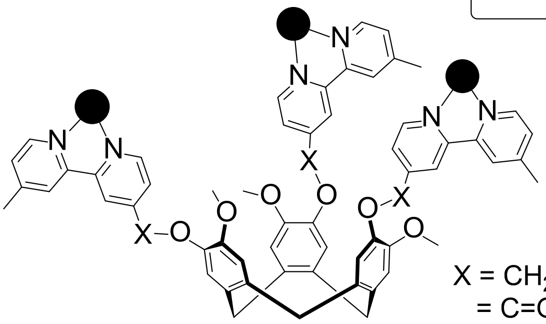

X = CH_2 complex **1.1**
= C=O complex **2.1**

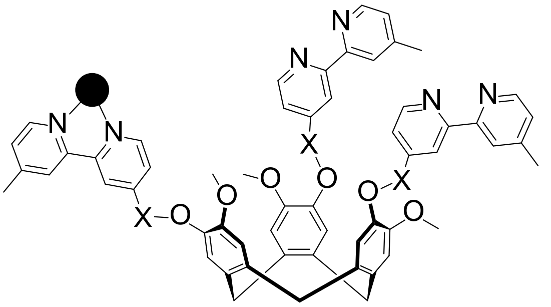

X = CH_2 complex **1.2**
= C=O complex **2.2**

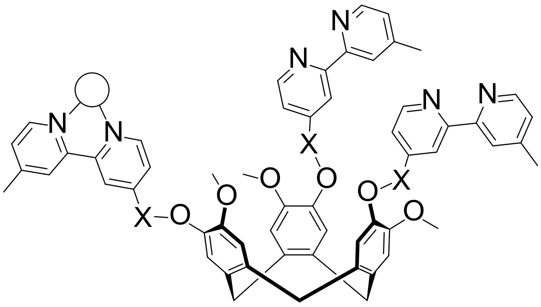

X = CH_2 complex **1.4a**
= C=O complex **2.4a**

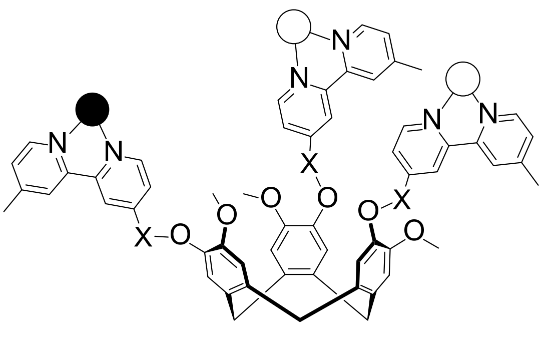

X = CH_2 complex **1.3**
= C=O complex **2.3**

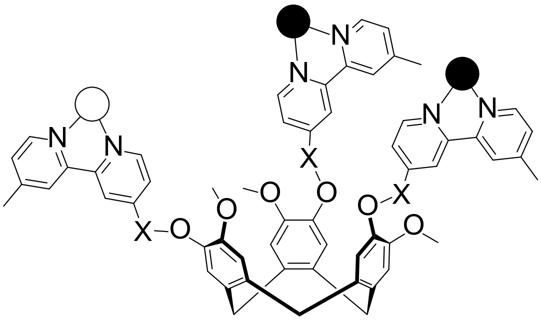

X = CH_2 complex **1.4**
= C=O complex **2.4**

Stereoisomers of $[\{Ir(ppy)_2\}_3L]^{3+}$ complexes

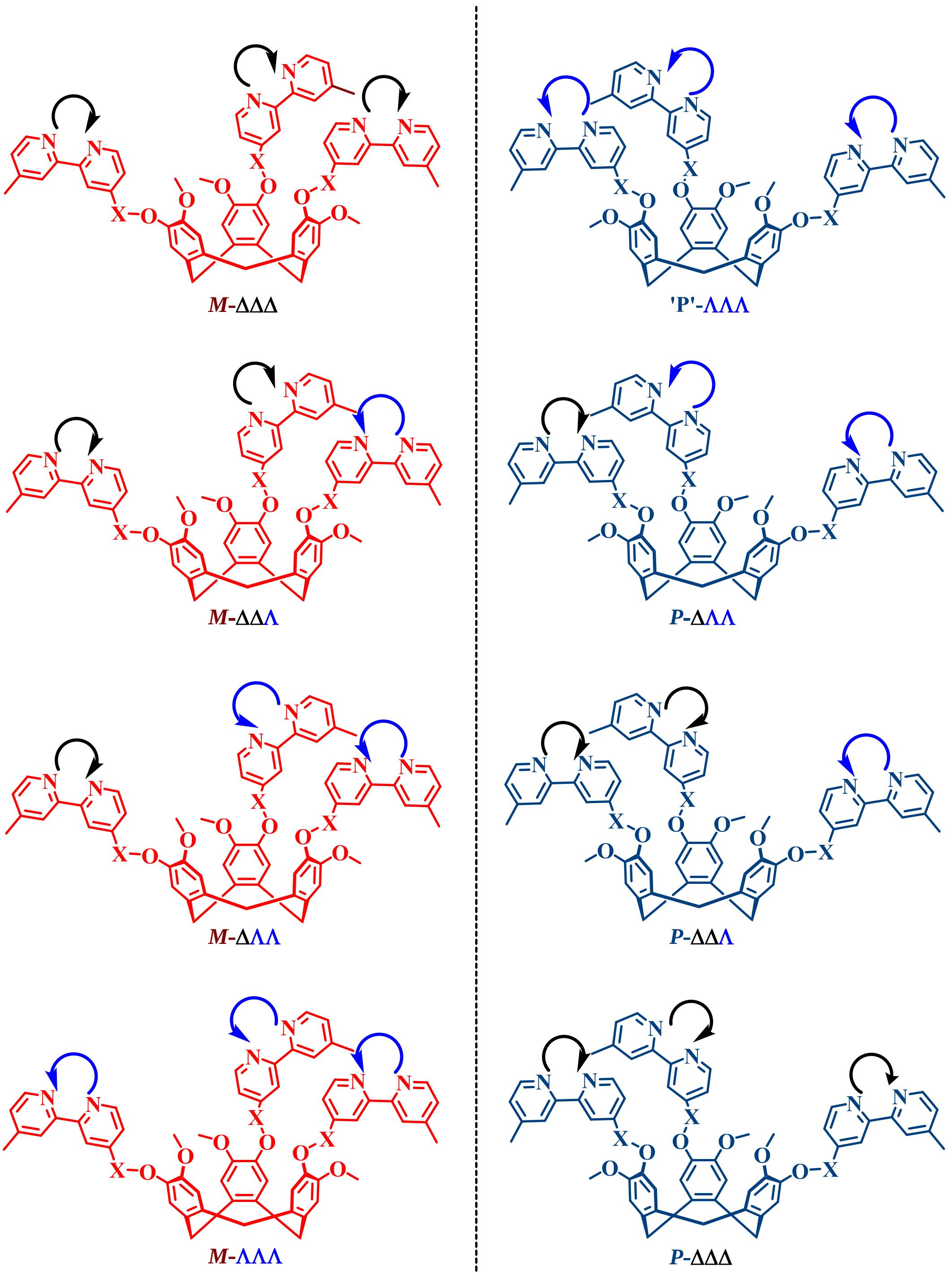

Figure S1: Possible stereoisomers of complexes 1.1. and 2.1; the M isomer shown in red and the P isomer in blue, with octahedral chirality represented by the curved arrows.

2. Mass Spectrometry

Complex 1.1

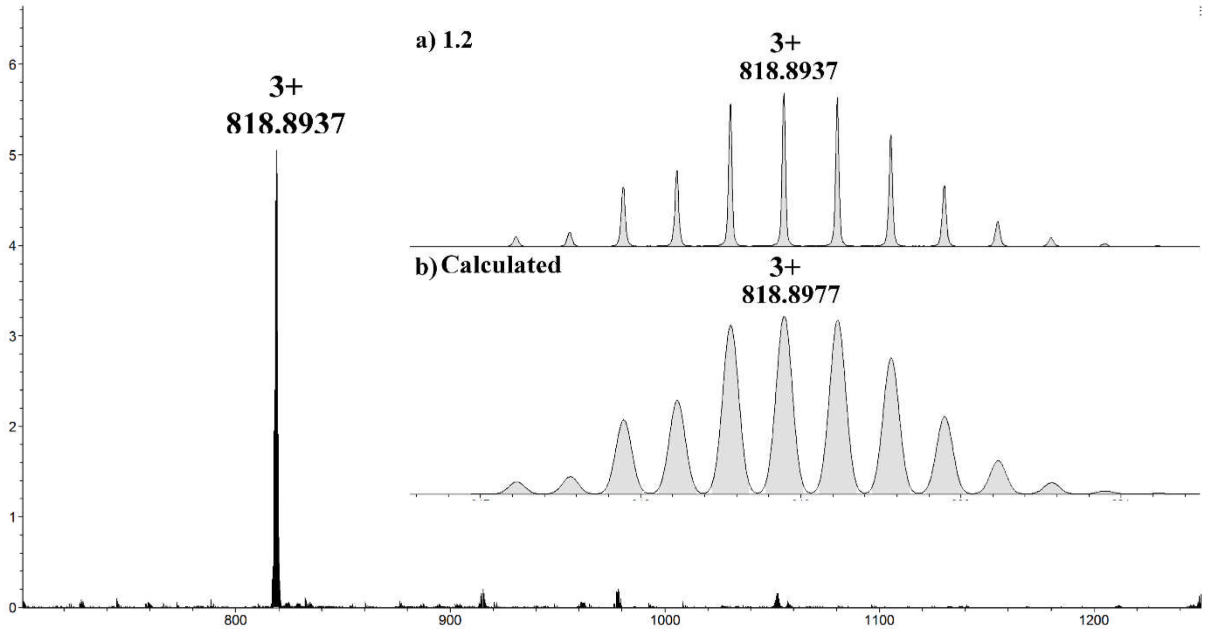

Figure S2: HR ESI-MS of 1.1 with inset showing A (observed) and B (calculated) isotopic distribution patterns.

Complex 1.2

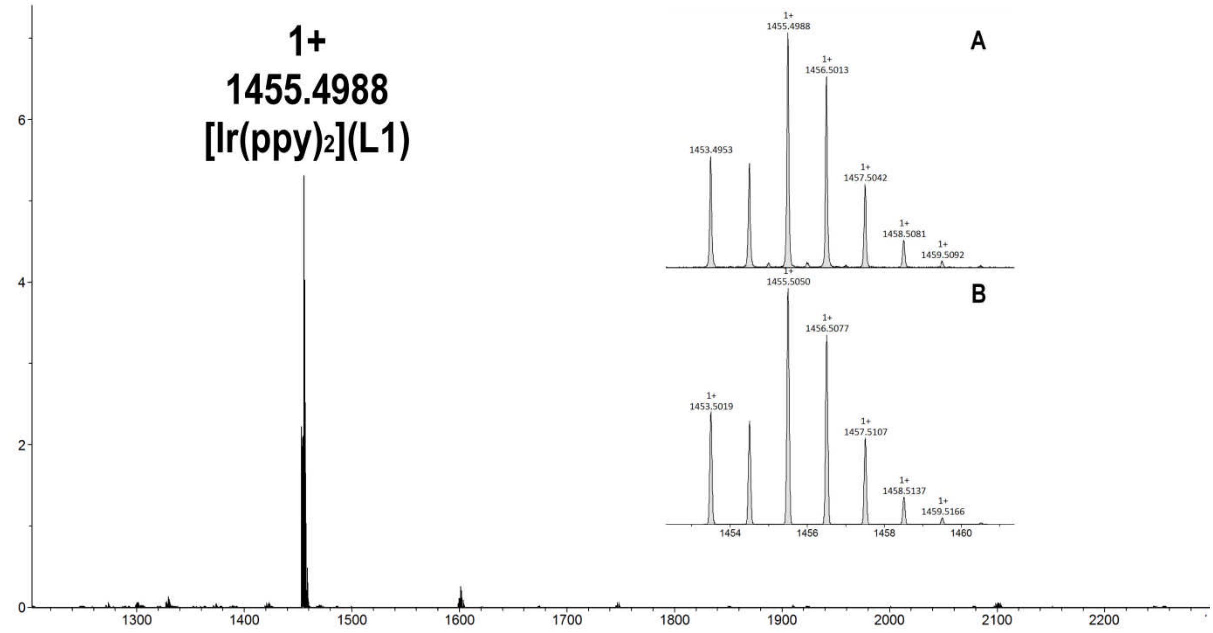

(a)

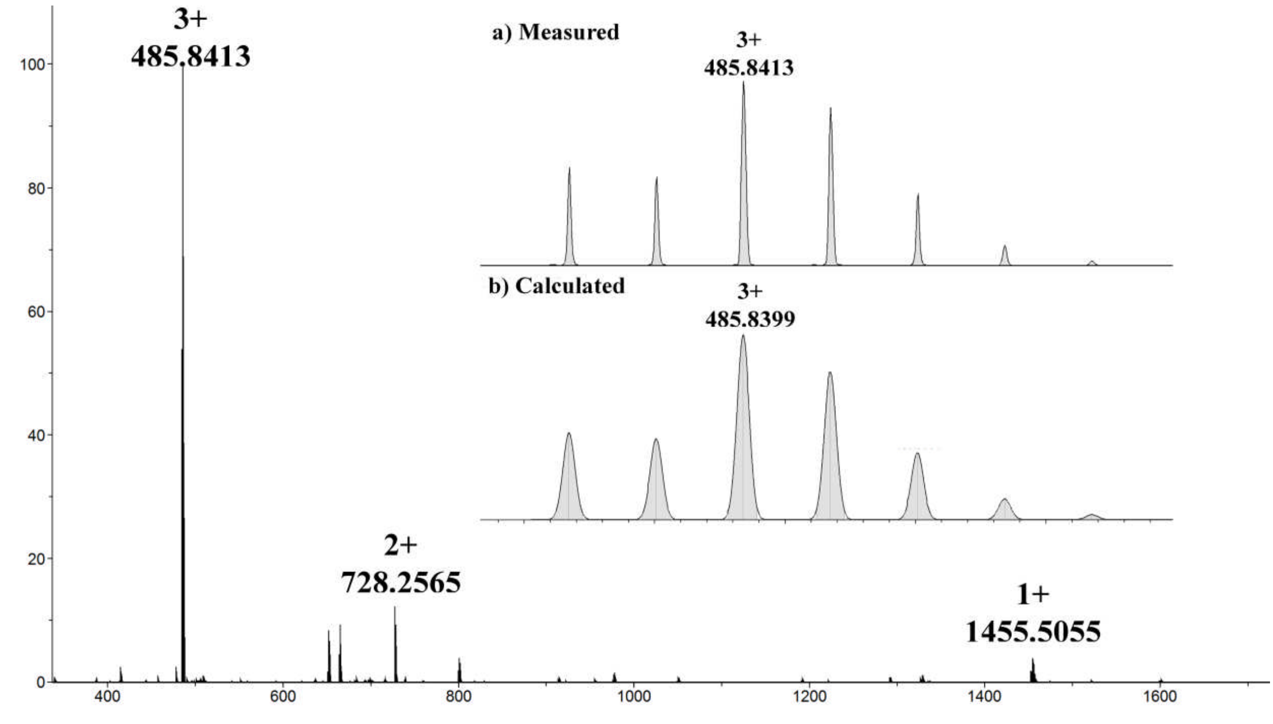

(b)

Figure S3: (a) HR ESI-MS of 1.2 with inset showing A (observed) and B (calculated) isotopic distribution patterns for $[Ir(ppy)_2L1]^+$ peak; (b) expanded spectrum also showing $\{[Ir(ppy)_2L1]\cdot 2H\}^{3+}$ (m/z 485) and $\{[Ir(ppy)_2L1]\cdot H\}^{2+}$ (m/z 728).

Complex 1.3

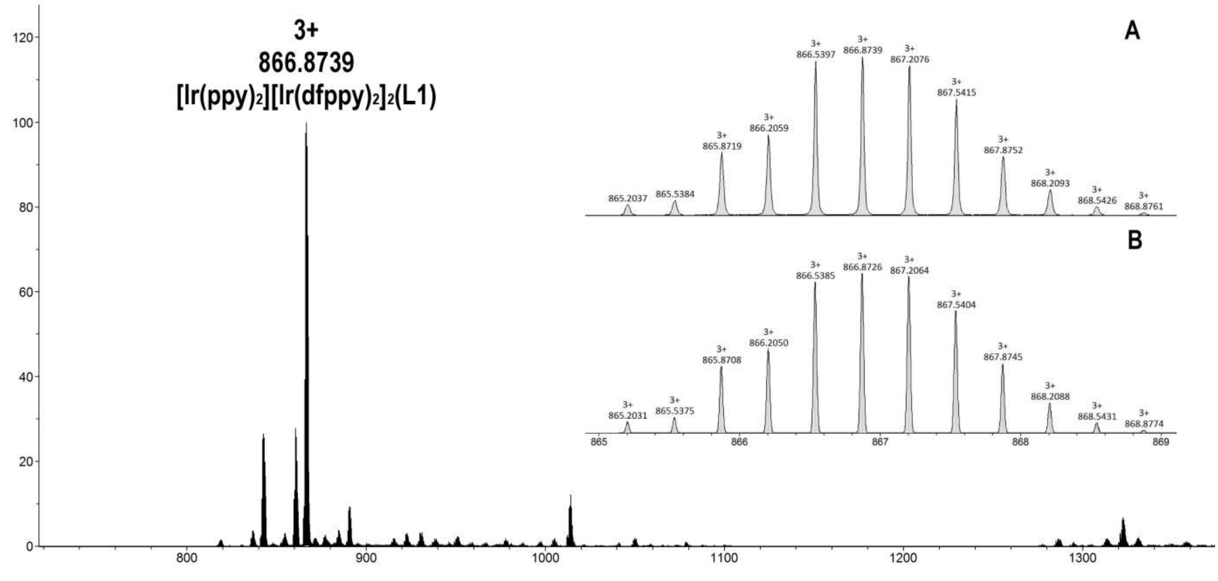

Figure S4: HR ESI-MS of 1.3 with inset showing A (observed) and B (calculated) isotopic distribution patterns.

Complex 1.4

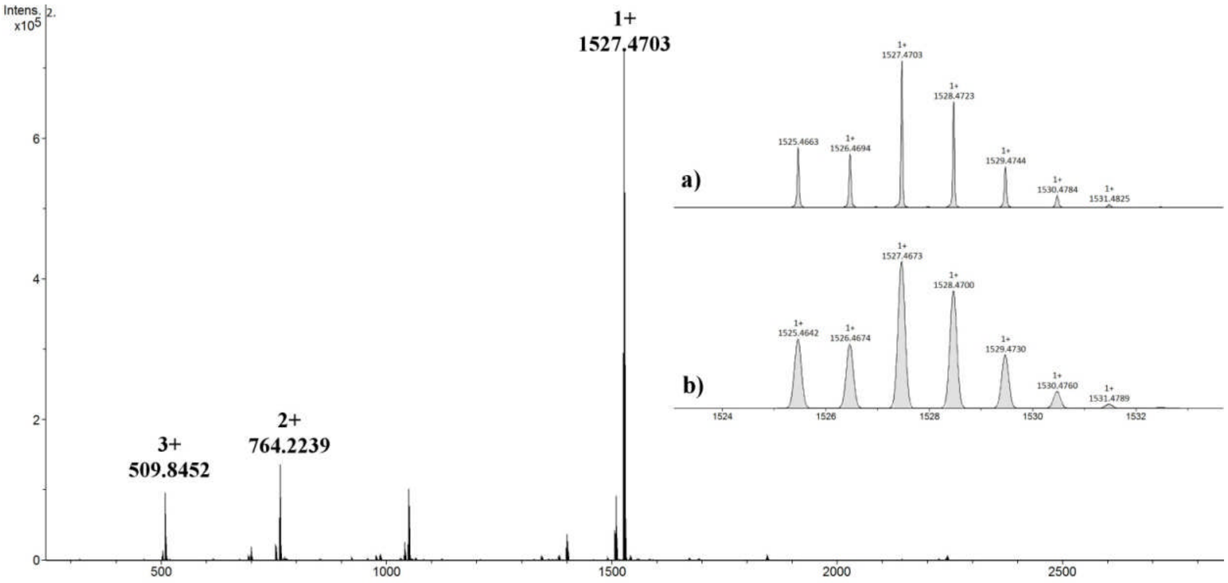

(a)

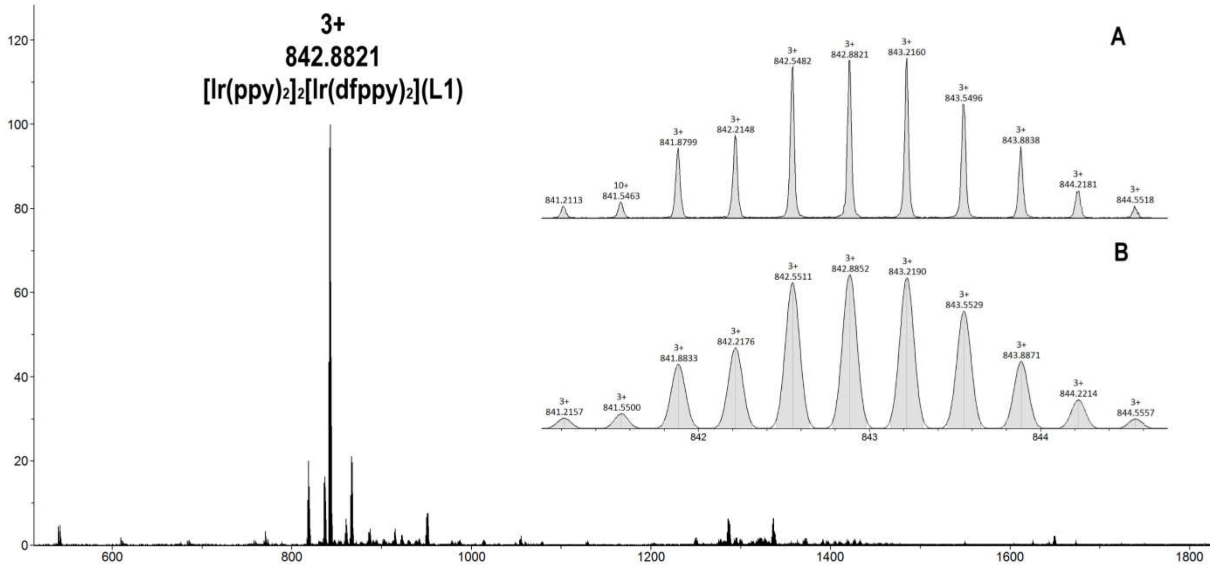

(b)

Figure S5: HR ESI-MS of (a) intermediate mono-metalled 1.4a showing [Ir(dFppy)$_2$L1]$^+$ at peak at m/z 1572 and peaks at m/z 509 and 764 corresponding to species {[Ir(dFppy)$_2$L1]·2H}$^{3+}$ and {[Ir(dFppy)$_2$L1]·H}$^{2+}$ respectively; (b) complex 1.4. Insets show A (observed) and B (calculated) isotopic distribution patterns.

Complex 2.1

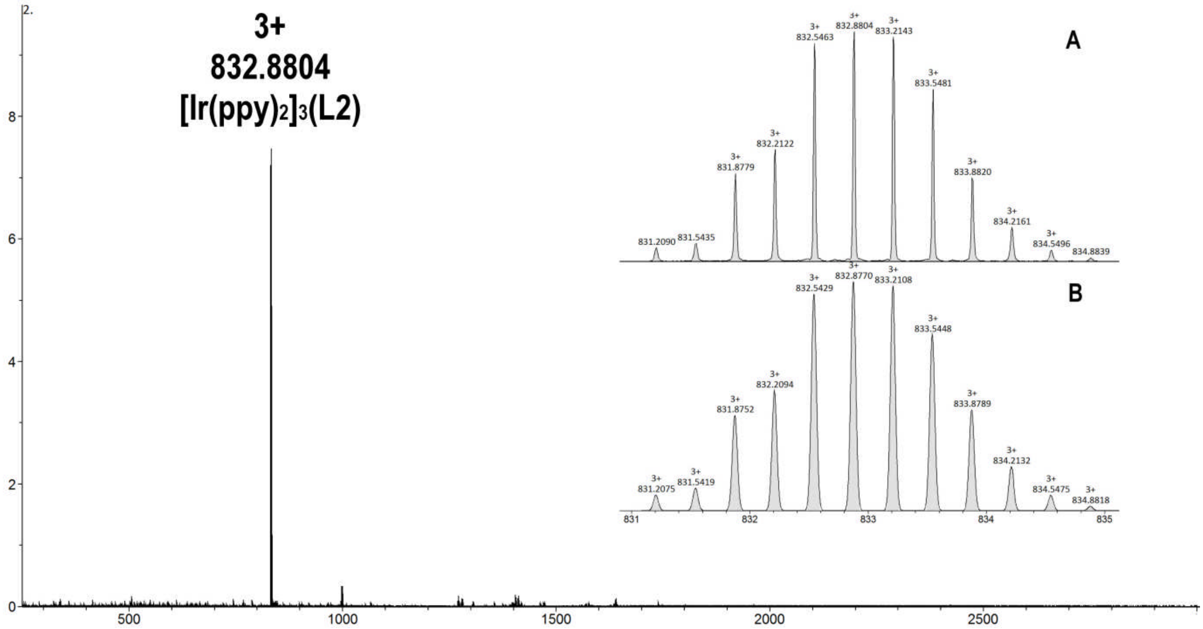

Figure S6: HR ESI-MS of 2.1 with inset showing A (observed) and B (calculated) isotopic distribution patterns.

Complex 2.2

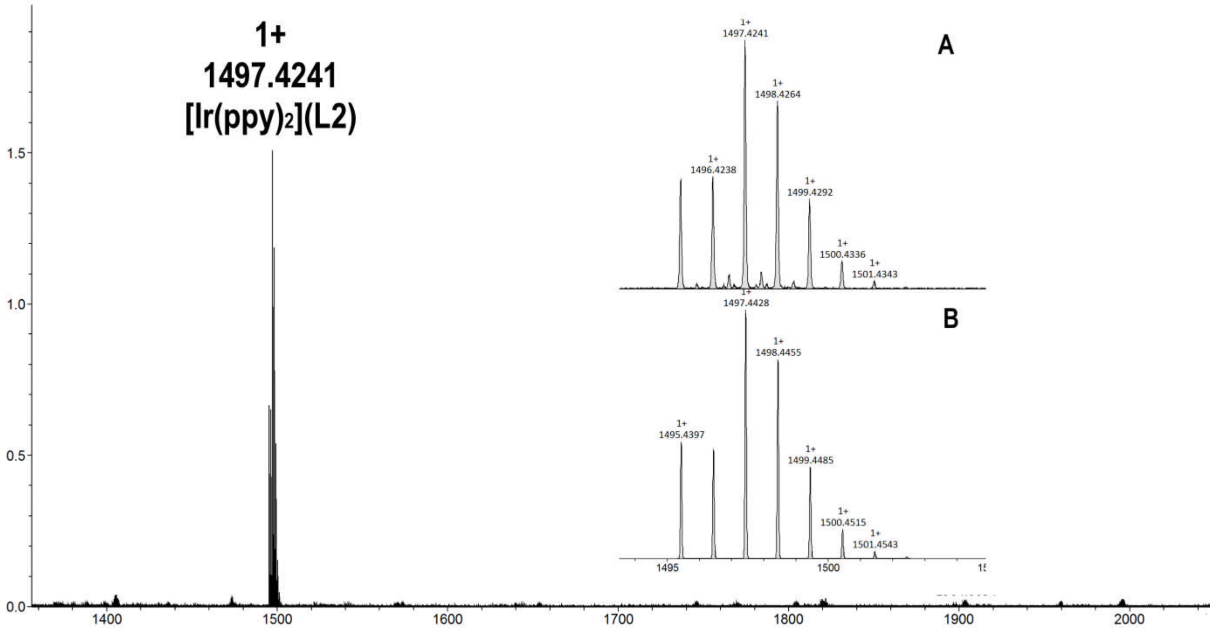

(a)

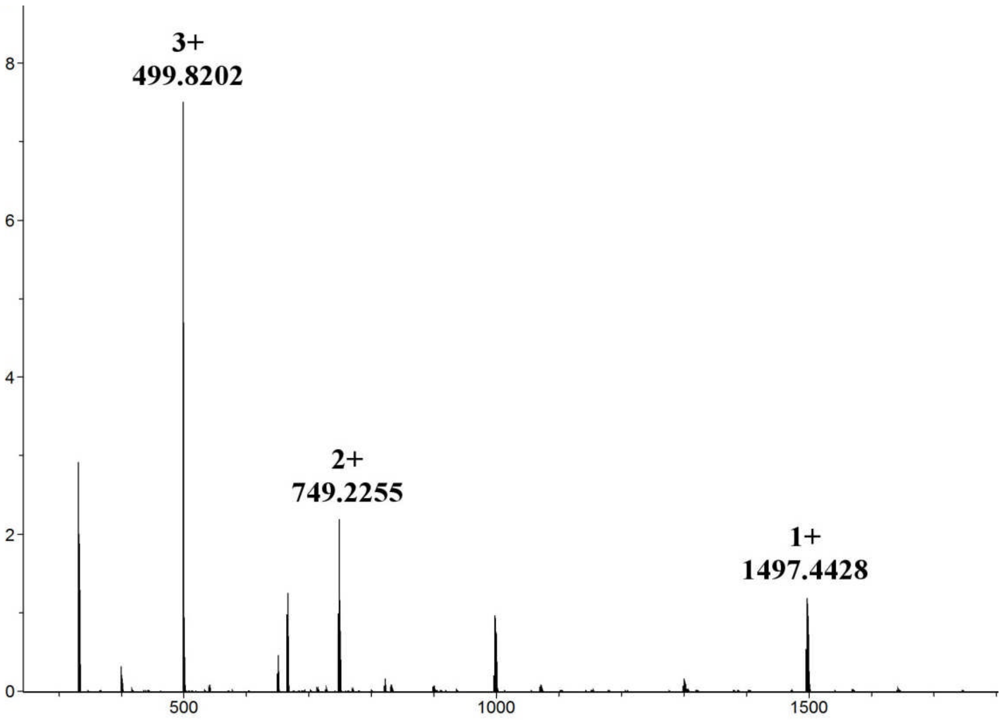

(b)

Figure S7: HR ESI-MS of (a) 2.2 with inset showing A (observed) and B (calculated) isotopic distribution patterns for $[Ir(ppy)_2L2]^+$ peak; (b) expanded spectrum also showing $\{[Ir(ppy)_2L2]\cdot 2H\}^{3+}$ (m/z 499) and $\{[Ir(ppy)_2L2]\cdot H\}^{2+}$ (m/z 749).

Complex 2.3

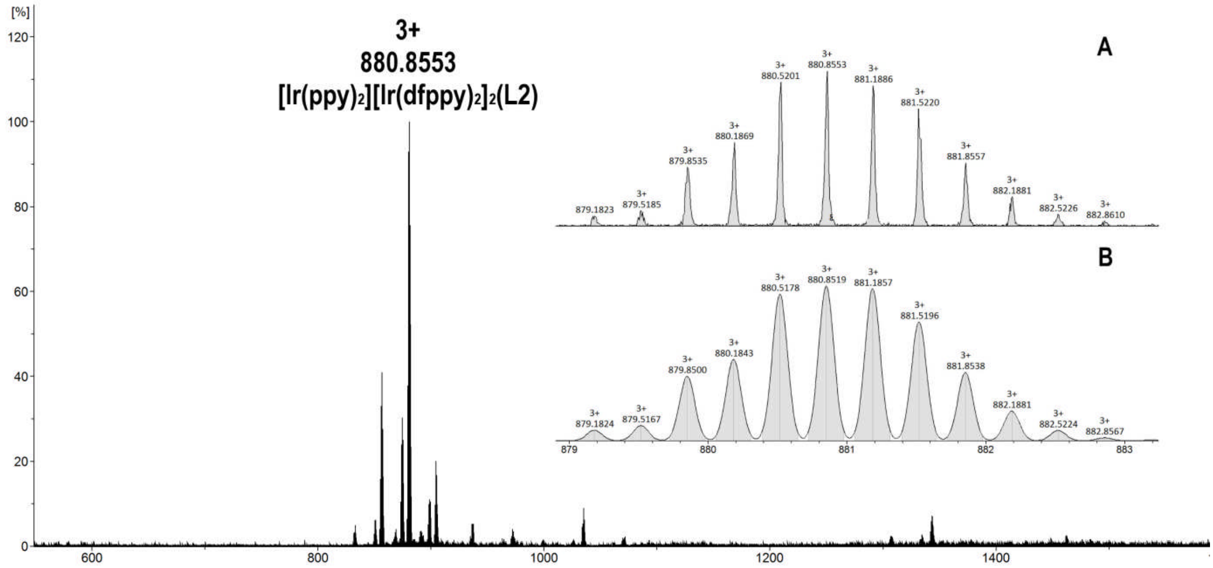

Figure S8: HR ESI-MS of 2.3 with with inset showing A (observed) and B (calculated) isotopic distribution patterns.

Complex 2.4

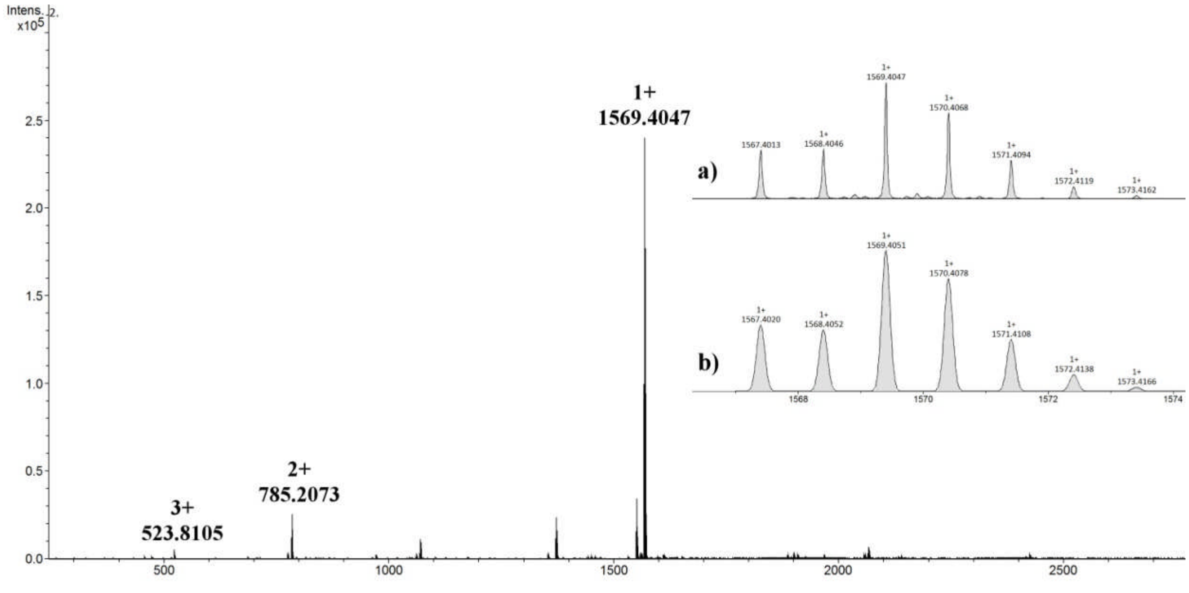

(a)

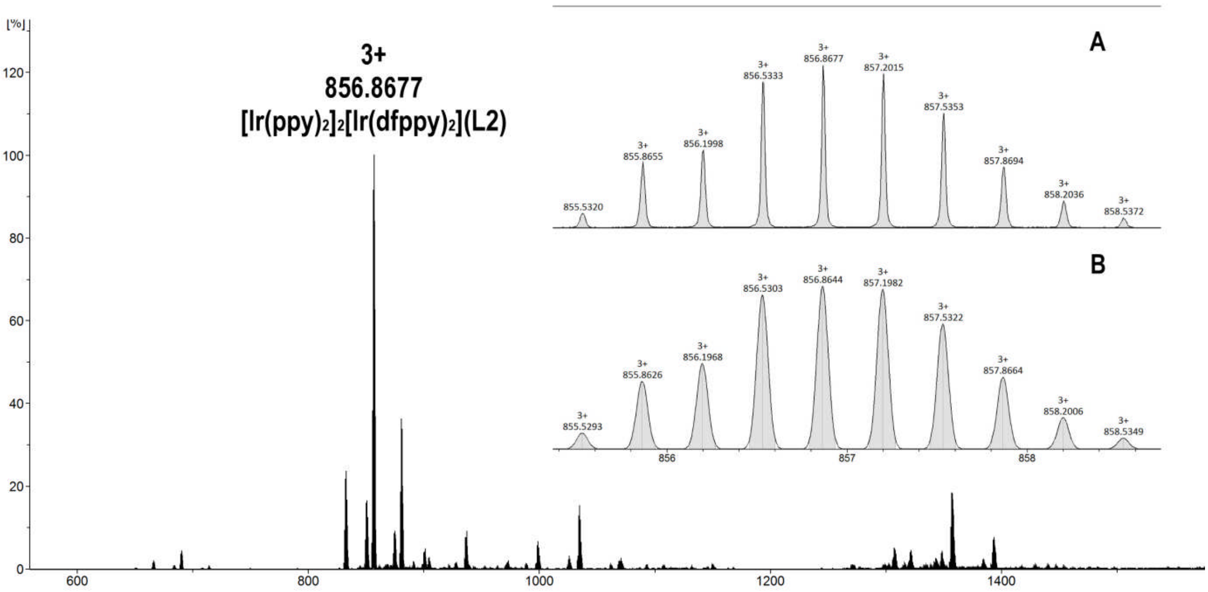

(b)

Figure S9: HR ESI-MS of (a) intermediate mono-metalled 2.4a showing $[Ir(dFppy)_2L2]^{+}$ at peak at m/z 1569 and peaks at m/z 523 and 785 corresponding to species $\{[Ir(dFppy)_2L2]\cdot 2H\}^{3+}$ and $\{[Ir(dFppy)_2L2]\cdot H\}^{2+}$ respectively (b) complex $\{[Ir(ppy)_2]_2[Ir(dFppy)_2](L2)\}^{3+}$ 2.4. Insets show A (observed) and B (calculated) isotopic distribution patterns.

3. NMR Spectroscopy

^{1}H NMR

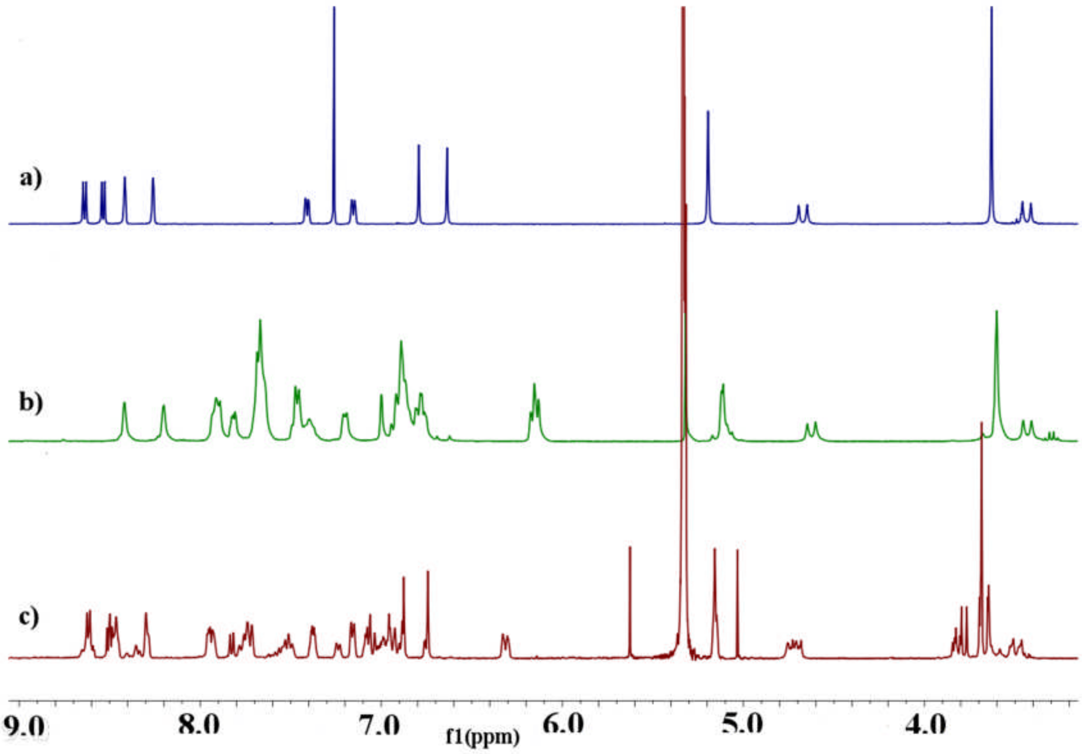

Figure S10: Comparative ^{1}H NMR spectra of a) L1 in d-$CHCl_3$ b) tri-metallic 1.1 in d_2-DCM c) mono-metallic 1.2 in d_2-DCM.

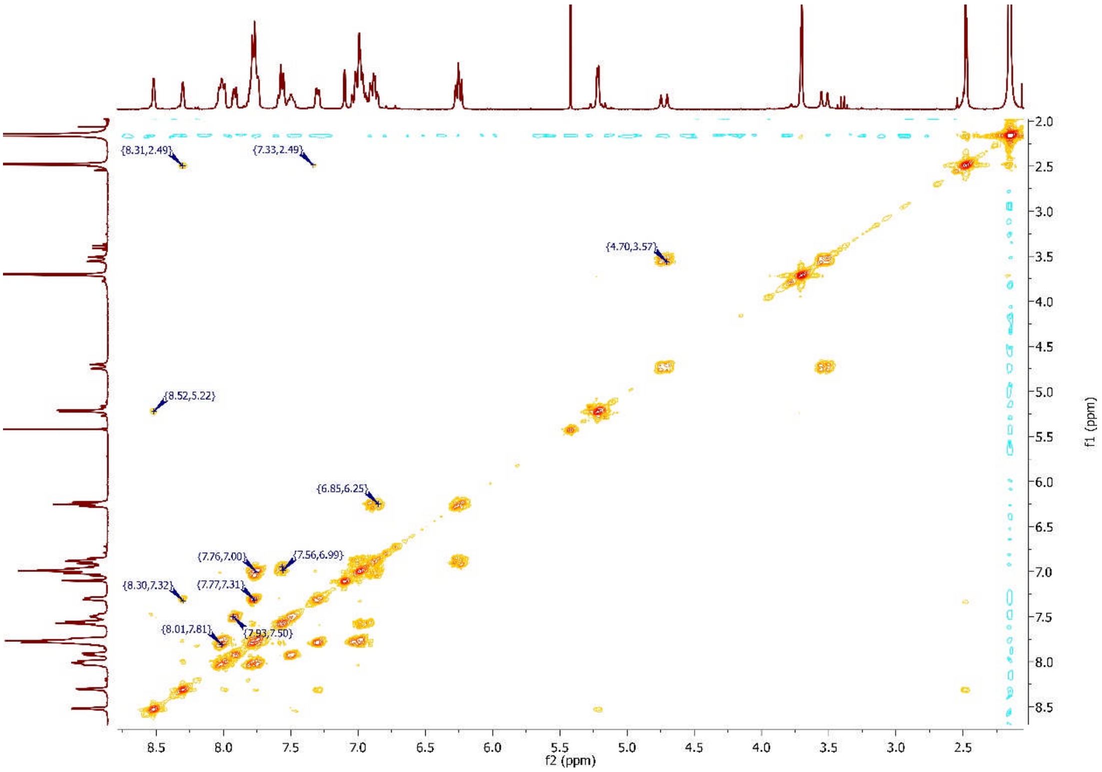

Figure S11: COSY NMR of complex 1.1.

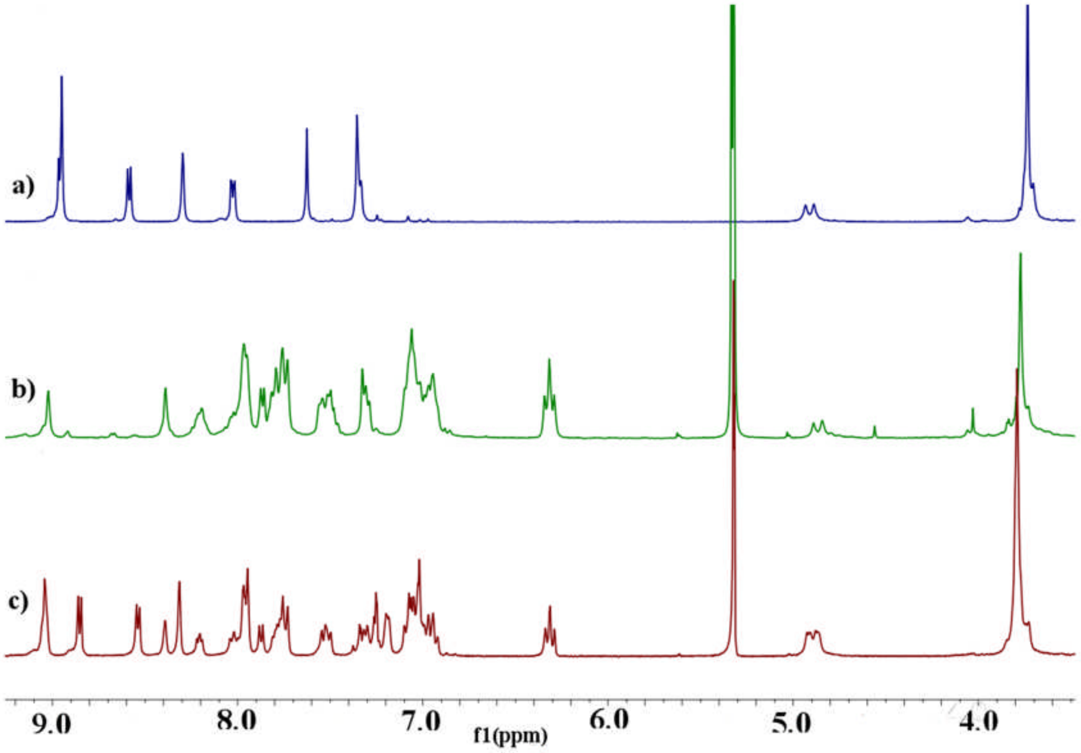

Figure S12: Comparative ^{1}H NMR spectra of a) L2 in d_6-DMSO b) tri-metallic 2.1 in d_2-DCM c) mono-metallic 2.2 in d_2-DCM.

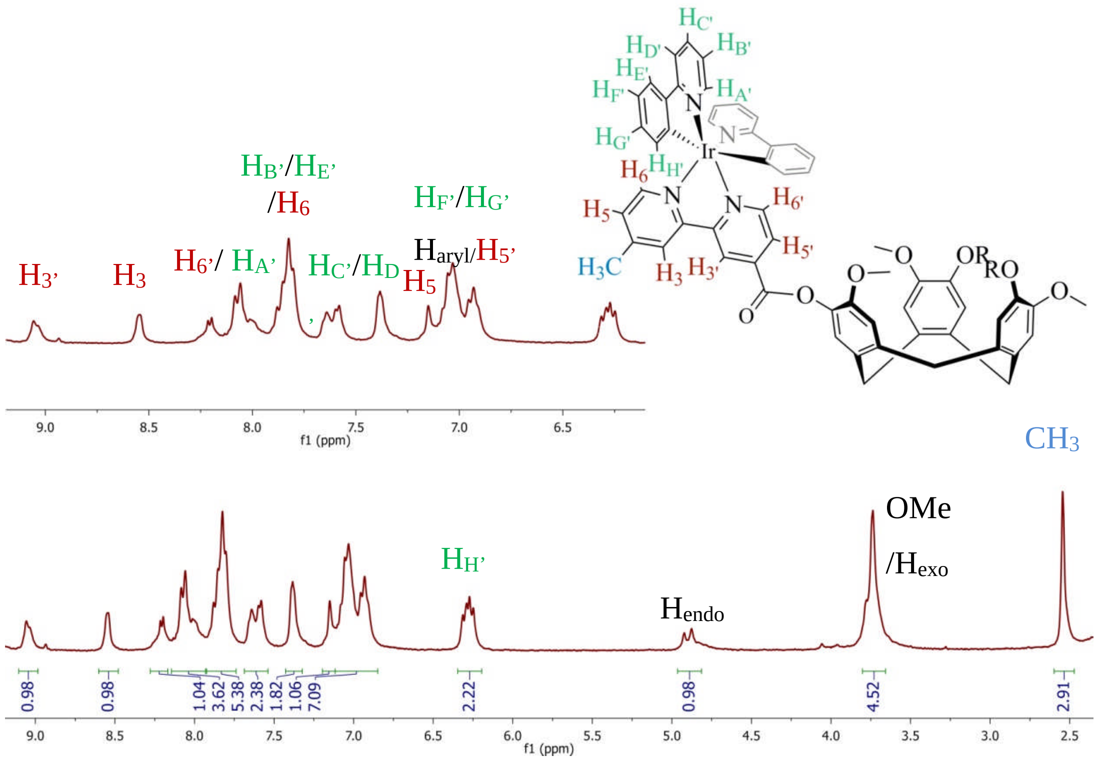

Figure S13: ¹H NMR (CD_3CN) of complex 2.1 showing peak assignments, only one the three equivalent $Ir(ppy)_2$ groups is shown for clarity.

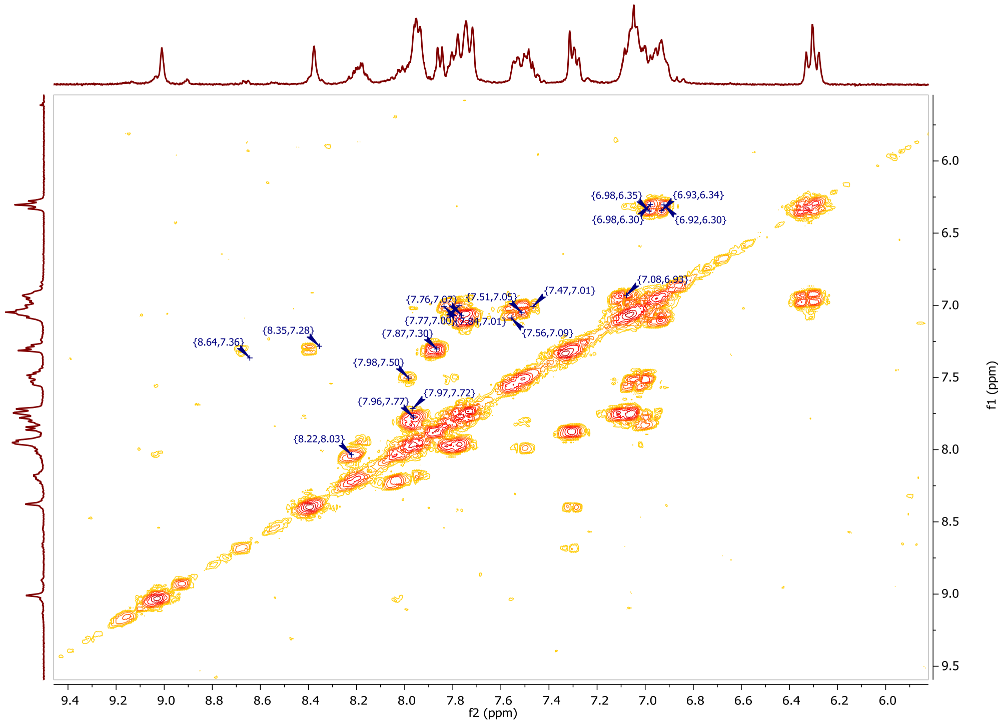

Figure S14: COSY NMR of complex 2.1.

Complex 1.3

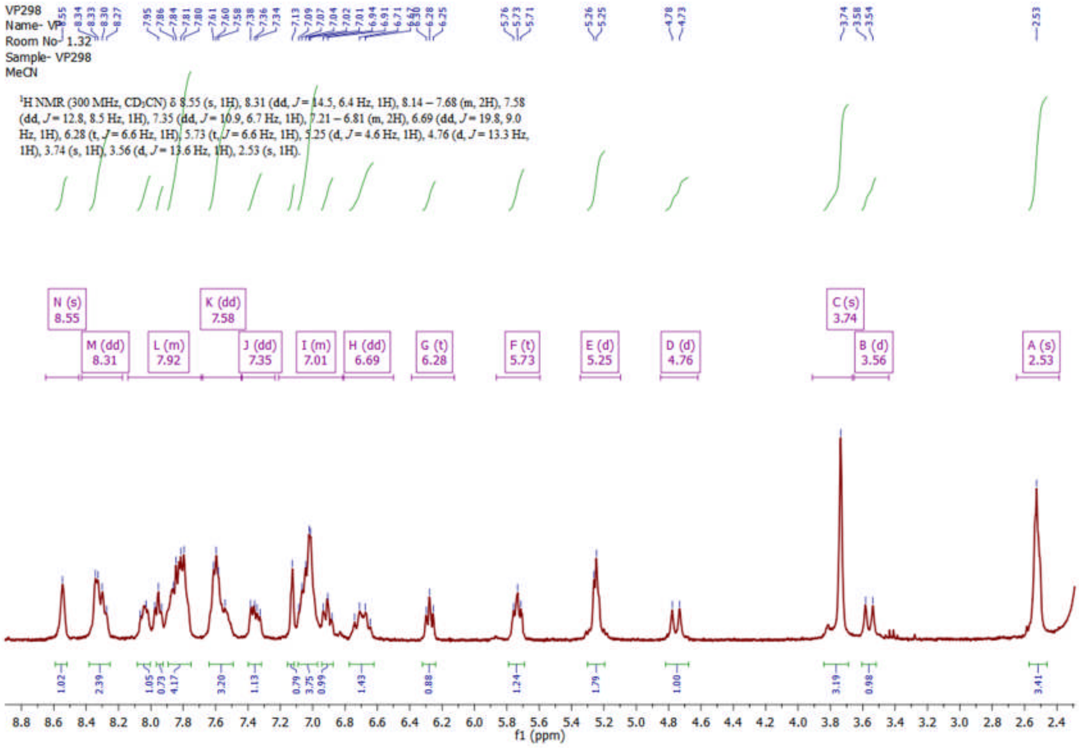

Figure S15: ^{1}H NMR Spectrum of 1.3 in d_3-MeCN.

Complex 1.4

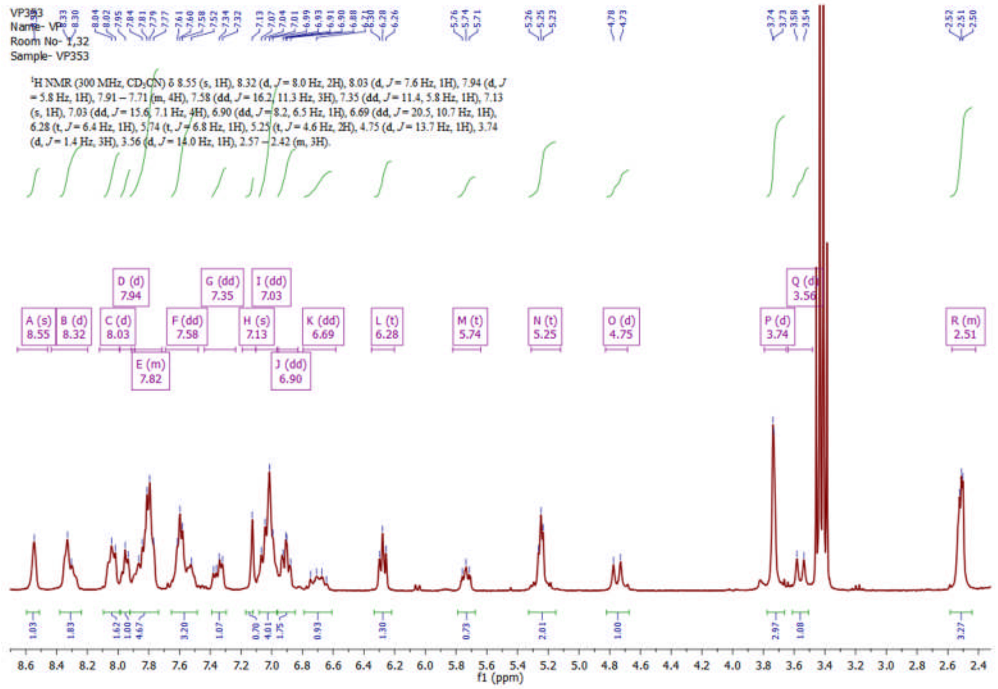

Figure S16: ^{1}H NMR Spectrum of complex 1.4 in d$_3$-MeCN.

Complex 2.3

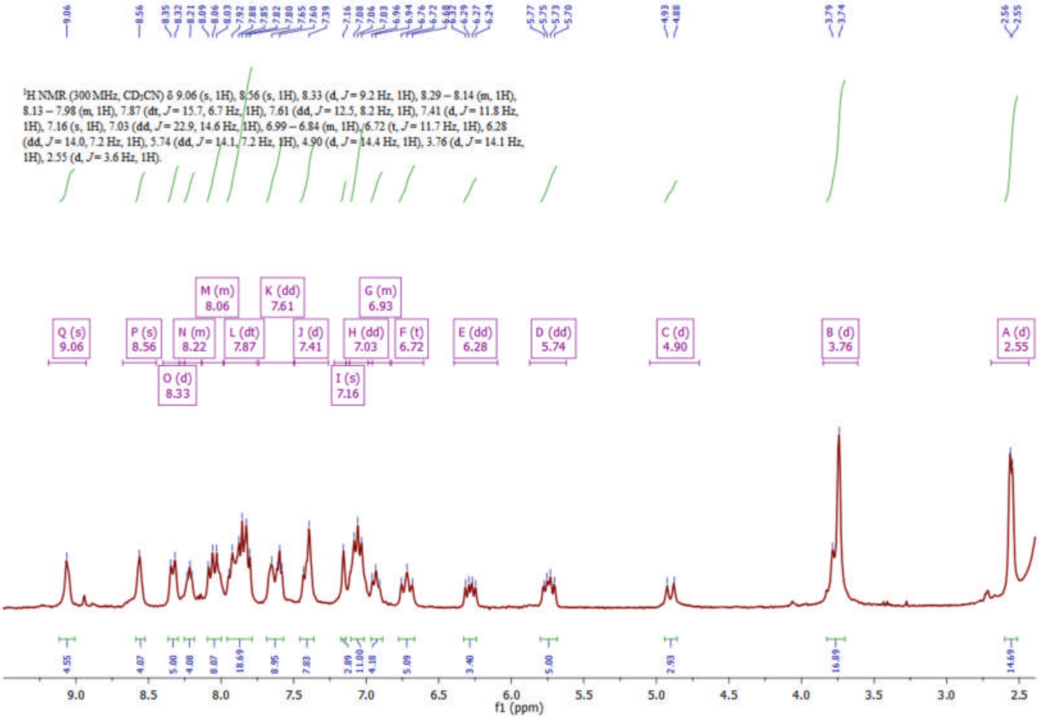

Figure S17: ^{1}H NMR Spectrum of 2.3 in d$_3$-MeCN.

Complex 2.4

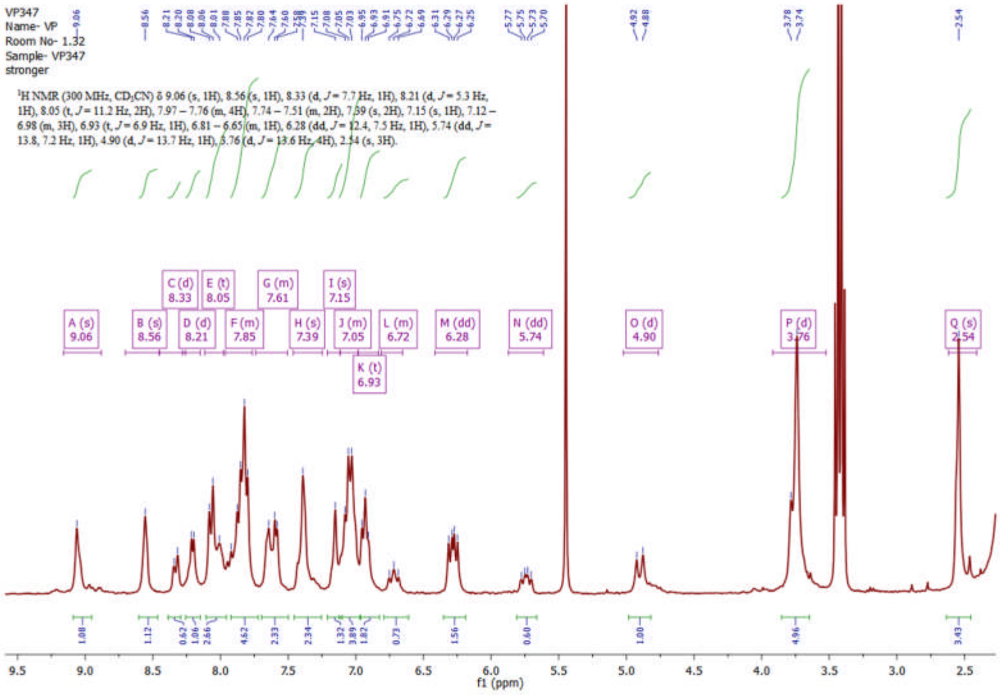

Figure S18: ^{1}H NMR spectrum of 2.4 in d_3-MeCN.

^{13}C NMR

Complex 1.1

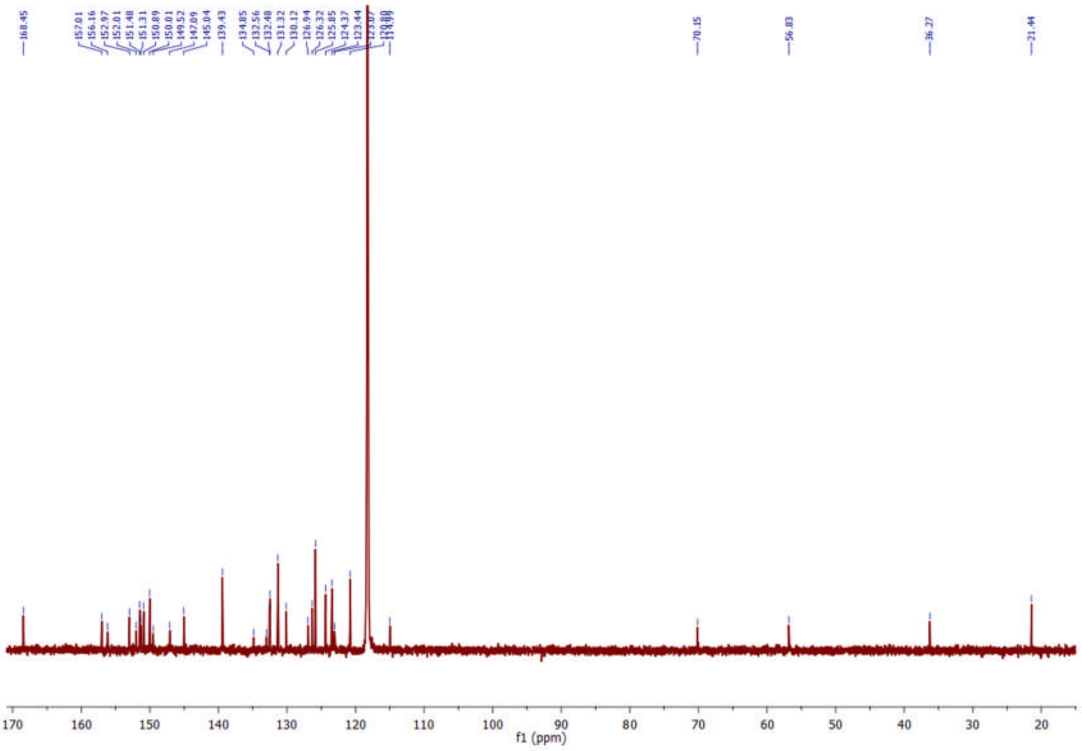

Figure S19: ***^{13}C NMR (75 MHz) spectrum of complex 1.1 in d_3-MeCN. ^{13}C NMR δ 168.43, 156.99, 156.15, 152.95, 151.96, 151.47, 151.41, 151.30, 150.88, 150.03, 150.00, 149.46, 147.06, 145.02, 144.96, 139.42, 134.81, 132.94, 132.54, 132.46, 131.31, 130.11, 126.93, 126.89, 126.31, 125.84, 124.36, 123.43, 123.10, 123.04, 120.79, 118.26, 114.94, 70.13, 56.78, 36.25, 21.44 ppm.***

Complex 1.2

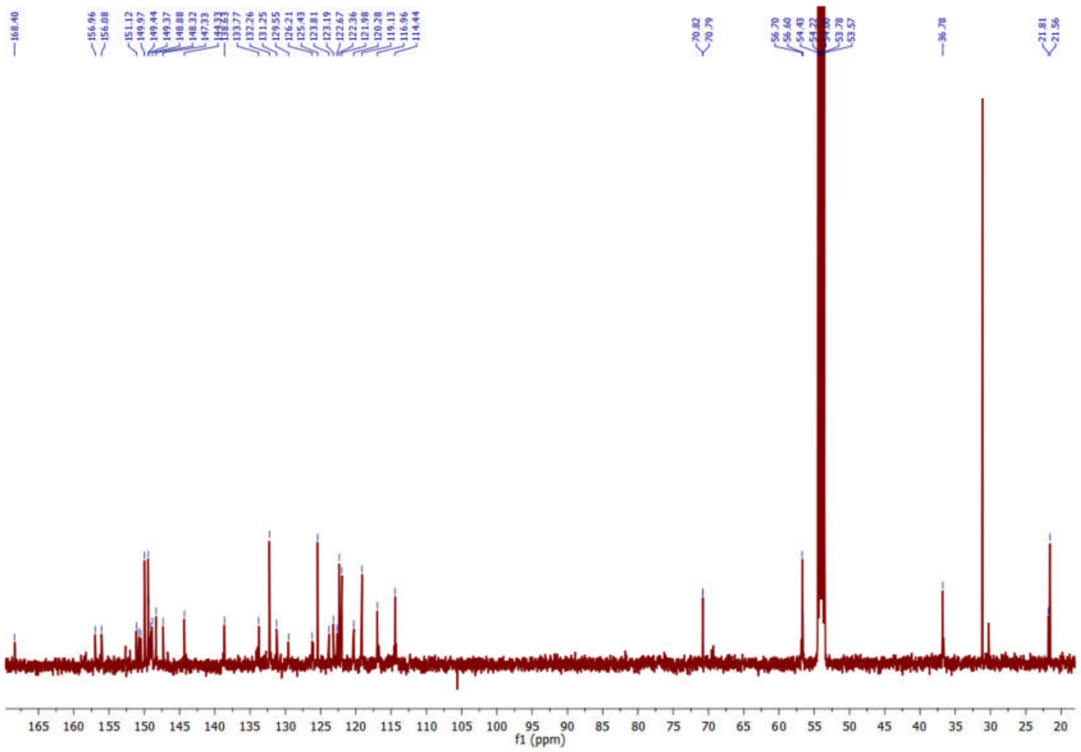

Figure S20: ^{13}C NMR (126 MHz) spectrum of 1.2 in d$_2$-DCM. δ 206.93, 156.96, 156.91, 156.11, 156.08, 151.12, 150.78, 150.49, 149.97, 149.96, 149.45, 149.37, 149.05, 148.88, 148.32, 147.33, 147.27, 144.33, 138.70, 138.63, 133.77, 132.26, 131.25, 131.23, 131.18, 129.55, 125.43, 125.41, 123.83, 123.80, 123.19, 123.14, 122.66, 122.36, 121.98, 120.39, 120.28, 119.13, 116.96, 114.44, 70.82, 70.79, 56.70, 56.60, 36.78, 31.15, 30.27, 21.81, 21.56 ppm.

Complex 1.3

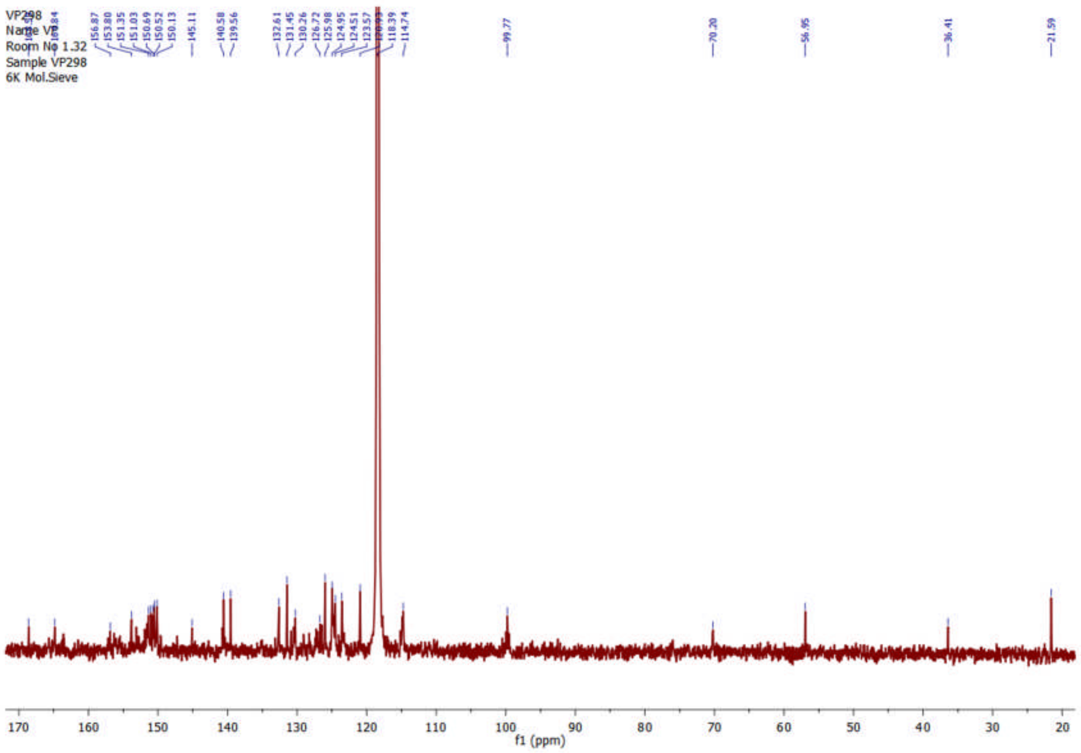

Figure S21: ***^{13}C NMR spectrum (126 MHz, CD_3CN) of 1.3 δ 191.60, 168.62, 164.89, 163.60, 161.44, 153.82, 153.11, 151.93, 151.55, 151.36, 150.53, 150.14, 147.31, 145.12, 140.74, 140.58, 139.57, 132.70, 132.63, 131.46, 130.88, 130.52, 130.27, 129.04, 128.26, 127.25, 127.05, 126.73, 126.46, 125.99, 124.96, 124.72, 124.50, 124.04, 123.58, 123.23, 120.94, 119.05, 115.17, 114.90, 114.82, 100.00, 99.78, 99.56, 70.24, 56.98, 36.42, 21.60 ppm***

Complex 1.4

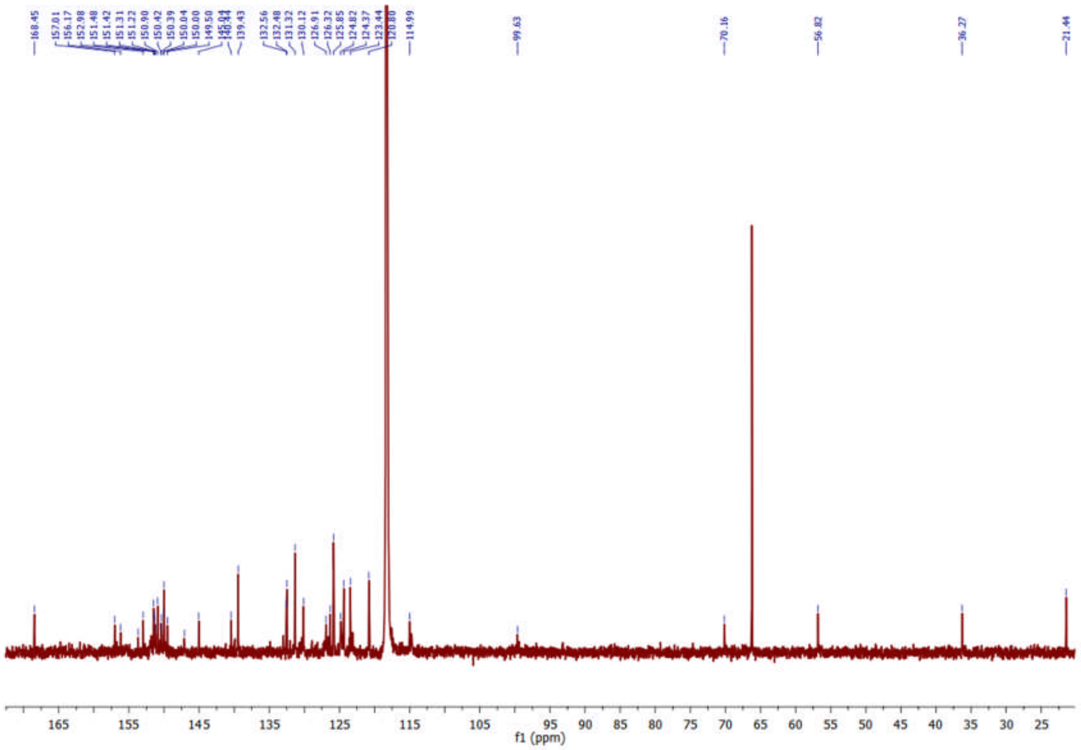

Figure S22: ***^{13}C NMR (126 MHz, CD_3CN) of complex 1.4. δ 168.45, 157.01, 156.17, 153.69, 152.98, 151.48, 151.42, 151.31, 151.22, 150.90, 150.42, 150.39, 150.04, 150.00, 149.50, 147.11, 145.04, 140.44, 139.43, 132.56, 132.48, 131.32, 130.12, 126.91, 126.32, 125.85, 124.82, 124.37, 123.44, 120.80, 114.99, 99.63, 70.16, 56.82, 36.27, 21.44 ppm.***

Complex 2.1

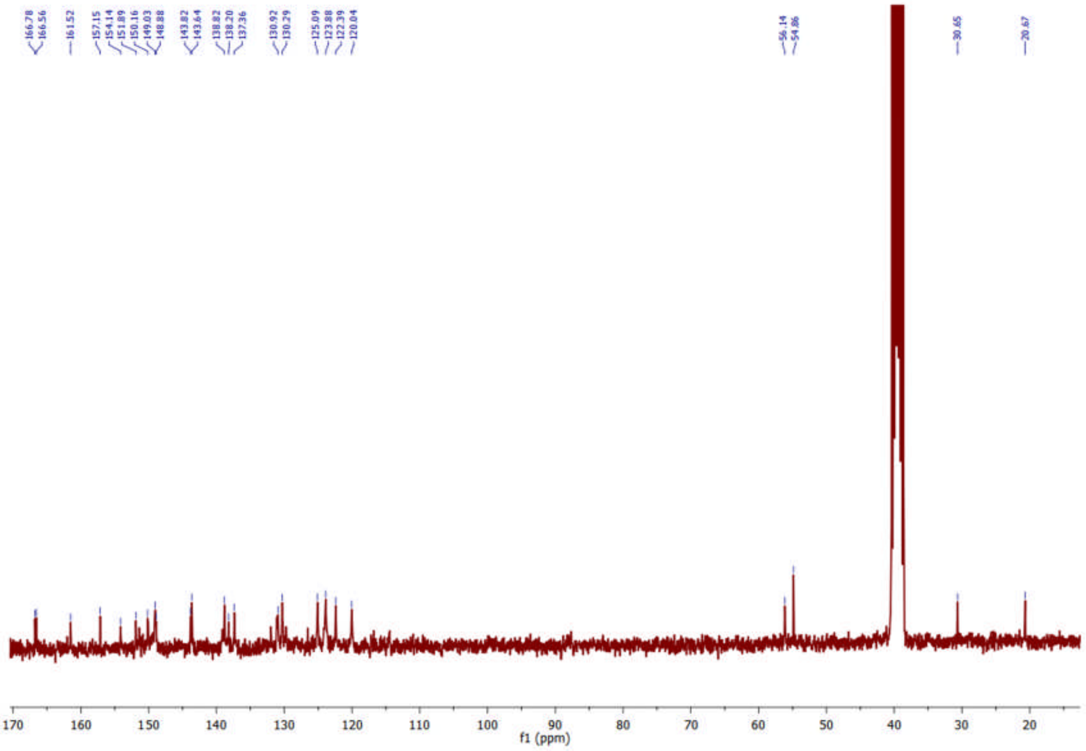

Figure S23: ^{13}C NMR (75 MHz, DMSO-d) spectrum of complex 2.1. δ 166.95, 166.72, 161.69, 157.32, 154.31, 152.05, 151.59, 151.57, 151.50, 150.32, 150.25, 150.22, 149.27, 149.19, 149.17, 149.08, 149.04, 143.98, 143.80, 139.00, 138.98, 138.94, 138.36, 137.52, 131.26, 131.08, 130.45, 126.71, 125.25, 125.23, 124.29, 124.09, 124.04, 122.55, 120.20, 56.31, 55.03, 30.81, 20.83 ppm.

Complex 2.2

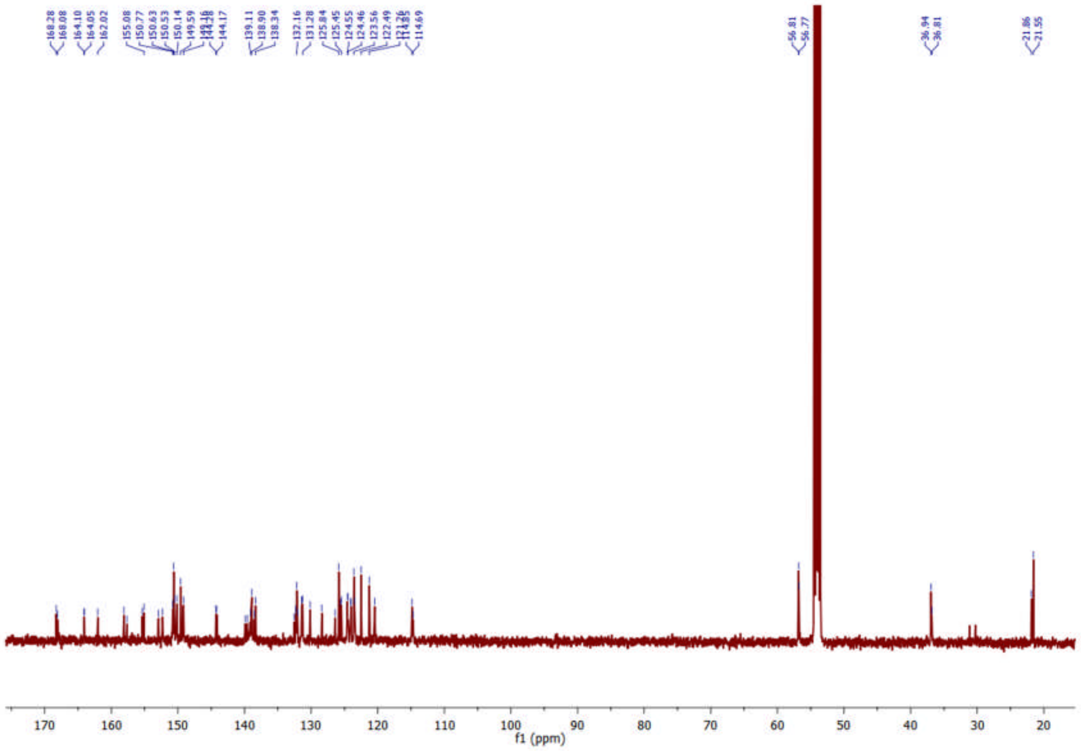

Figure S24: ^{13}C NMR (126 MHz) spectrum of 2.2 in d$_2$-DCM. δ 168.28, 168.08, 164.10, 164.09, 164.05, 162.02, 158.11, 158.09, 158.08, 157.67, 157.64, 155.37, 155.08, 152.92, 152.91, 152.31, 150.77, 150.63, 150.53, 150.18, 150.14, 149.59, 149.27, 149.18, 149.16, 149.13, 144.28, 144.17, 139.89, 139.86, 139.58, 139.57, 139.17, 139.11, 139.01, 139.00, 138.97, 138.93, 138.89, 138.51, 138.34, 132.54, 132.33, 132.30, 132.20, 132.17, 132.16, 132.04, 131.41, 131.28, 130.15, 128.38, 126.40, 126.39, 125.84, 125.82, 125.55, 125.45, 124.57, 124.55, 124.49, 124.46, 124.08, 124.00, 123.99, 123.56, 123.48, 123.40, 122.49, 122.46, 121.26, 121.23, 120.49, 120.44, 114.91, 114.86, 114.85, 114.69, 56.85, 56.81, 56.77, 36.93, 36.81, 21.86, 21.55 ppm.

Complex 2.3

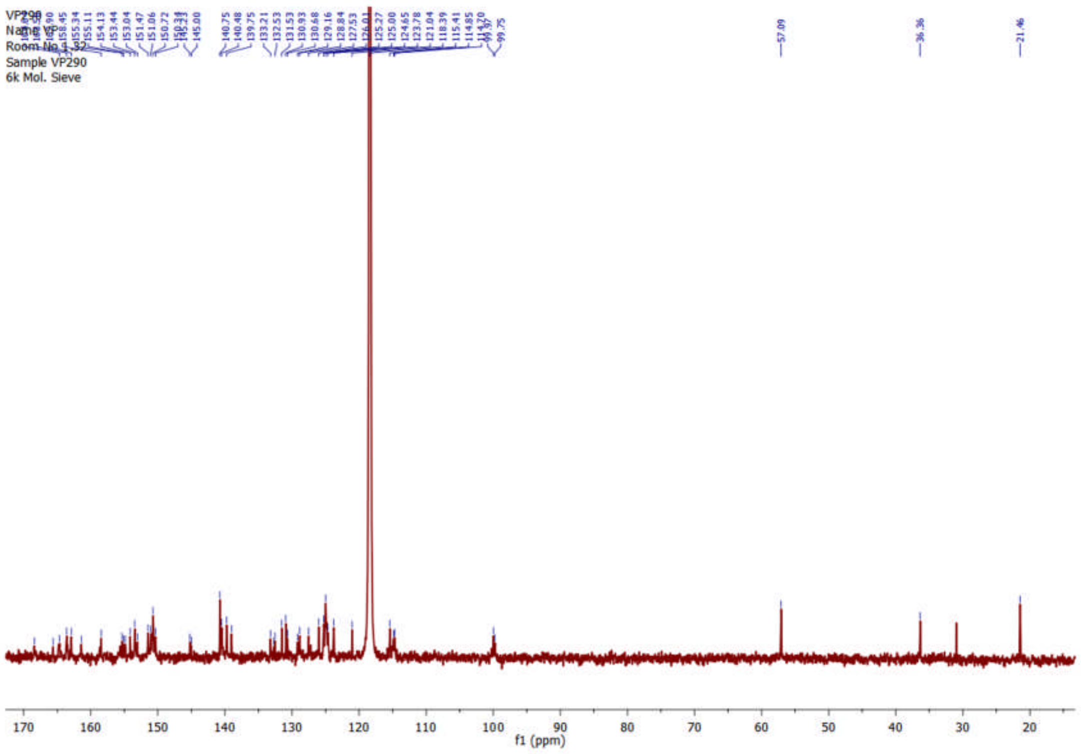

Figure S25: ***^{13}C NMR (126 MHz) spectrum of 2.3 in d_3-MeCN. δ 168.26, 164.83, 163.43, 162.91, 162.77, 161.39, 155.49, 153.96, 153.31, 151.23, 150.53, 150.19, 145.11, 144.87, 140.62, 140.56, 140.35, 139.62, 138.89, 132.58, 132.40, 131.38, 130.79, 130.65, 130.55, 128.70, 127.39, 127.24, 127.09, 125.88, 125.01, 124.87, 124.79, 124.52, 123.66, 120.91, 115.28, 114.71, 114.56, 105.95, 100.06, 99.83, 56.95, 36.22, 21.31 ppm.***

Complex 2.4

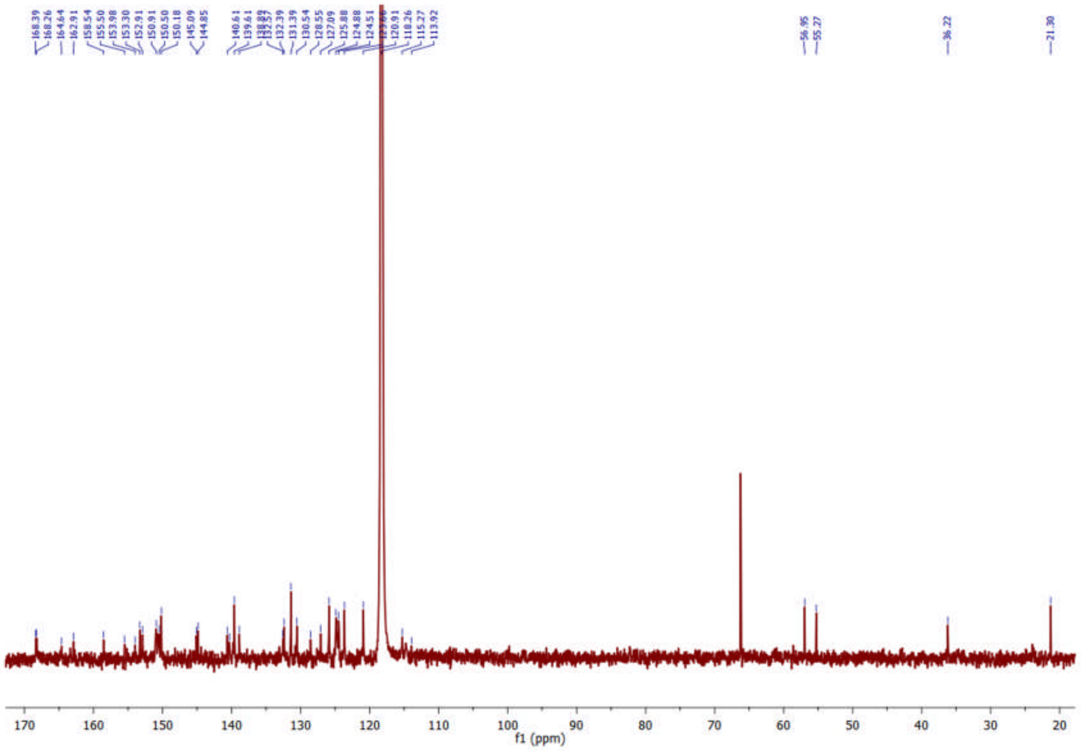

Figure S26: ***^{13}C NMR(126 MHz) spectrum of 2.4 in d_3-MeCN. δ 168.39, 168.26, 164.64, 162.91, 158.54, 155.50, 153.98, 153.30, 152.91, 150.91, 150.50, 150.18, 145.09, 144.85, 140.61, 140.32, 139.61, 138.89, 132.57, 132.39, 131.39, 130.54, 128.55, 127.09, 125.88, 124.88, 124.51, 123.66, 120.91, 118.26, 115.27, 113.92, 56.95, 36.22, 21.30 ppm.***

4. Photophysical Measurements

All samples were prepared in HPLC grade acetonitrile with varying concentrations in the order of $10^{-4} - 10^{-6}$ M. Absorption spectra were recorded at room temperature using a Shimadzu UV-1800 double beam spectrophotometer. Molar absorptivity determination was verified by linear least-squares fit of values obtained from at least four independent solutions at varying concentrations with absorbance ranging from 6.05×10^{-5} to 2.07×10^{-5} M.

The sample solutions for the emission spectra were prepared in HPLC-grade MeCN and degassed *via* freeze-pump-thaw cycles using a quartz cuvette designed in-house. Steady-state emission and excitation spectra and time-resolved emission spectra were recorded at 298 K using an Edinburgh Instruments F980. All samples for steady-state measurements were excited at 360 nm, while samples for time-resolved measurements were excited at 378 nm using a PDL 800-D pulsed diode laser. Emission quantum yields were determined using the optically dilute method.[1] A stock solution with absorbance of *ca*. 0.5 was prepared and then four dilutions were prepared with dilution factors between 2 and 20 to obtain solutions with absorbances of *ca*. 0.095 0.065, 0.05 and 0.018, respectively. The Beer-Lambert law was found to be linear at the concentrations of these solutions. The emission spectra were then measured after the solutions were rigorously degassed *via* three freeze-pump-thaw cycles prior to spectrum acquisition. For each sample, linearity between absorption and emission intensity was verified through linear regression analysis and additional measurements were acquired until the Pearson regression factor (R^2) for the linear fit of the data set surpassed 0.9. Individual relative quantum yield values were calculated for each solution and the values reported represent the slope value. The equation $\Phi_s = \Phi_r(A_r/A_s)(I_s/I_r)(n_s/n_r)^2$ was used to calculate the relative quantum yield of each of the sample, where Φ_r is the absolute quantum yield of the reference, n is the refractive index of the solvent, A is the absorbance at the excitation wavelength, and I is the integrated area under the corrected emission curve. The subscripts s and r refer to the sample and reference, respectively. A solution of quinine sulfate in 0.5 M H_2SO_4 (Φ_r = 54.6%) [2] was used as external references.[3]

UV-visible

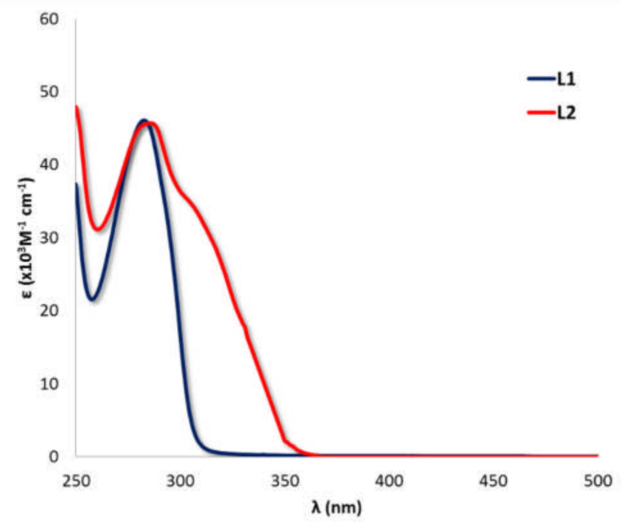

Figure S27. UV-Vis spectra of ligands L1, blue line and L2 (red line) were collected in MeCN at 298K.

Table **S1**. [a]UV-Vis absorption in MeCN at 298 K. [b] concentration-independent extinction coefficients.

**Complex**	**λ_{max}(vis)**[a] [ε(×10^3 M^{-1} cm^{-1})][b]
**1.1**	254 [189.4], 297 [110.9], 310 [80.4], 340 [36.3], 377 [23.3], 417 [7.7], 469 [1.7]
**1.2**	275 [91.6], 316 [22.0], 345 [12.0], 384 [7.29], 424 [6.2], 473 [1.5]
**2.1**	255 [170.6], 263 [153.6], 291 [113.3], 388 [28.6], 500 [1.9]
**2.2**	272 [85.3], 285 [82.9], 389 [11.8], 500 [1.8]
**1.3**	264 [155.2], 297 [92.2], 310 [71.1], 364 [20.9], 413 [8.5], 465 [1.8]
**1.4**	263 [155.8], 295 [92.9], 310 [71.5], 364 [20.9], 414 [8.6], 465 [1.6]
**2.3**	268 [135.5], 288 [100.9], 317 [70.5], 364 [26.4], 477 [1.8]
**2.4**	269 [122.6], 291 [89.9], 314 [60.6], 366 [24.4], 495 [1.6]

Emission properties

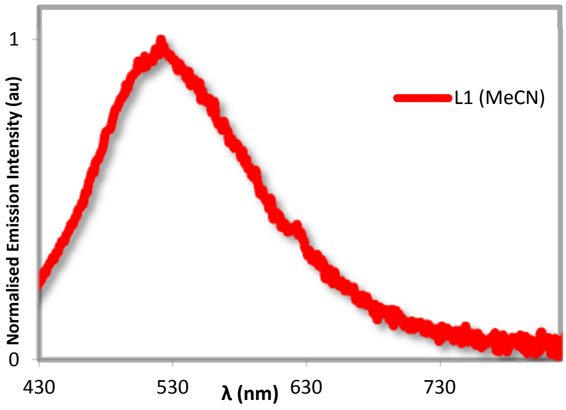

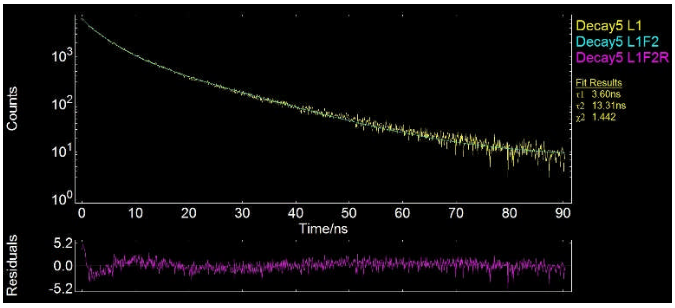

Figure S28. Left: Normalised photoluminescence spectra of ligand L1 collected in MeCN at 298 K upon photoexcitation at 360 nm. Right: Lifetime decays of L1 after excitation at 379 nm in degassed MeCN at 298 K (λ_{em} = 520 nm).

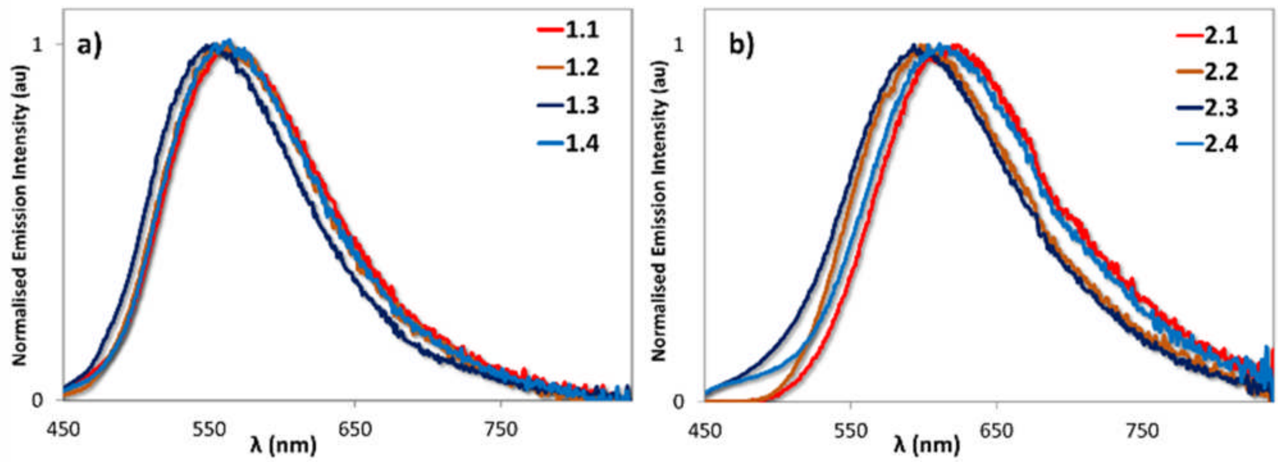

Figure S29. Normalised photoluminescence spectra of a) 1.1, red line; 1.2, orange line; 1.3, blue line and 1.4, light-blue line; b) 2.1, red line; 2.2, orange line; 2.3, blue line and 2.4, light-blue line. The spectra were collected in PMMA-doped films with 5 wt % of complexes spin-coated on a quartz substrate at 298 K upon photoexcitation at 360 nm.

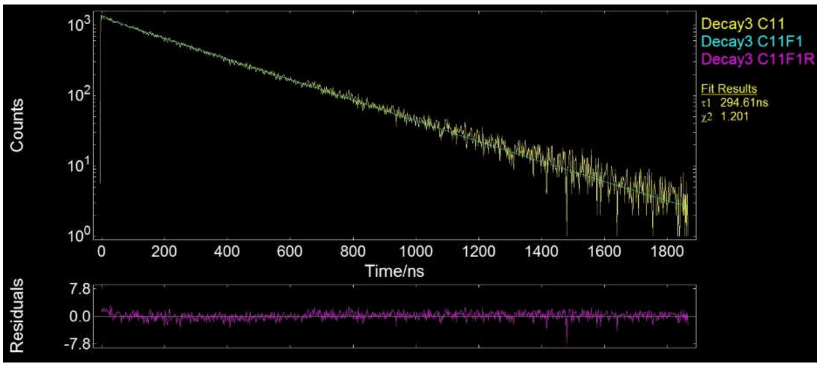

Figure S30. Lifetime decays of 1.1 after excitation at 379 nm in degassed MeCN at 298 K (λ_{em} = 610 nm).

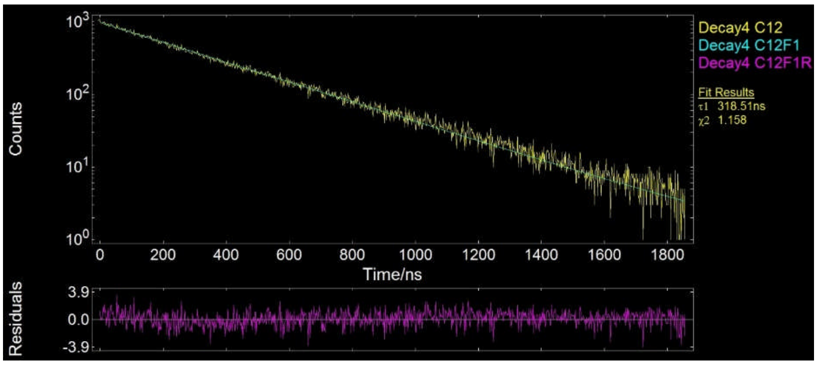

Figure S31. Lifetime decays of 1.2 after excitation at 379 nm in degassed MeCN at 298 K (λ_{em} = 608 nm).

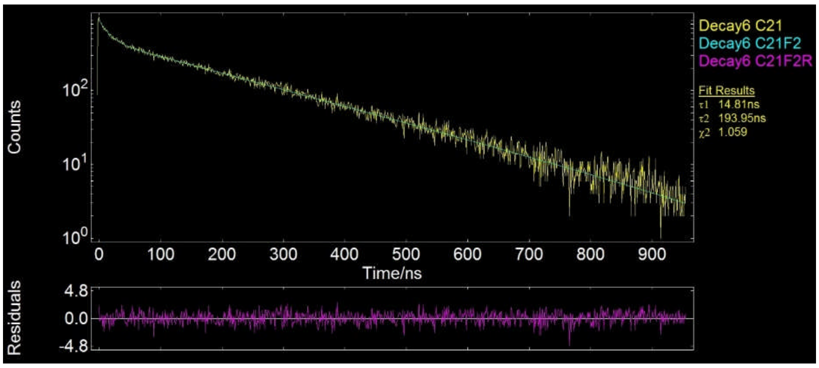

Figure S32. Lifetime decays of 2.1 after excitation at 379 nm in degassed MeCN at 298 K (λ_{em} = 686 nm).

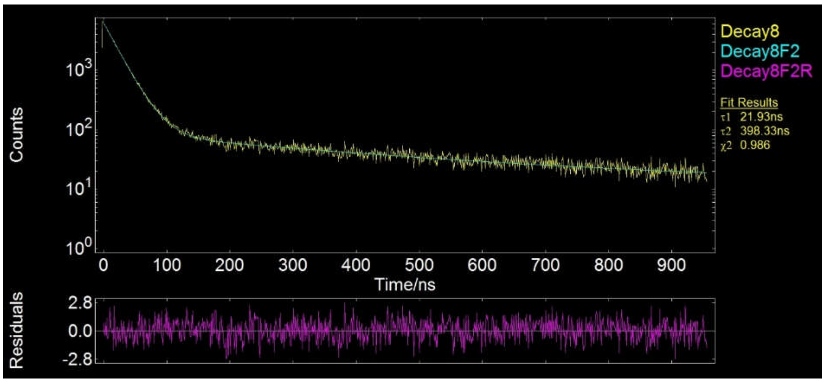

Figure S33. Lifetime decays of 2.2 after excitation at 379 nm in degassed MeCN at 298 K (λ_{em} = 686 nm).

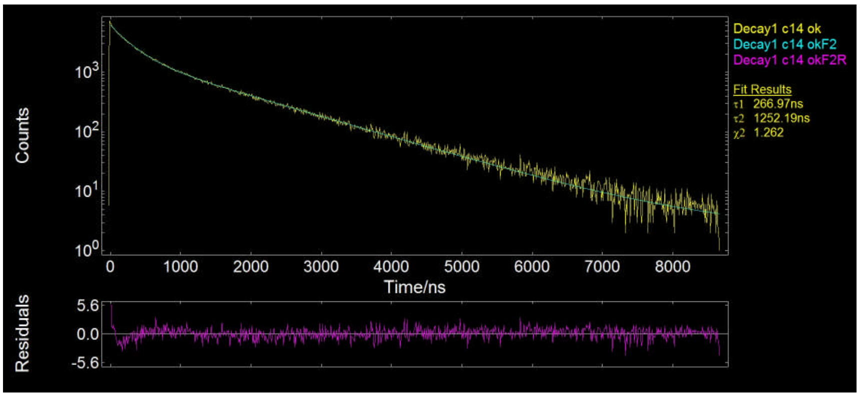

Figure S34. Lifetime decays of 1.3 after excitation at 379 nm in degassed MeCN at 298 K (λ_{em} = 574 nm).

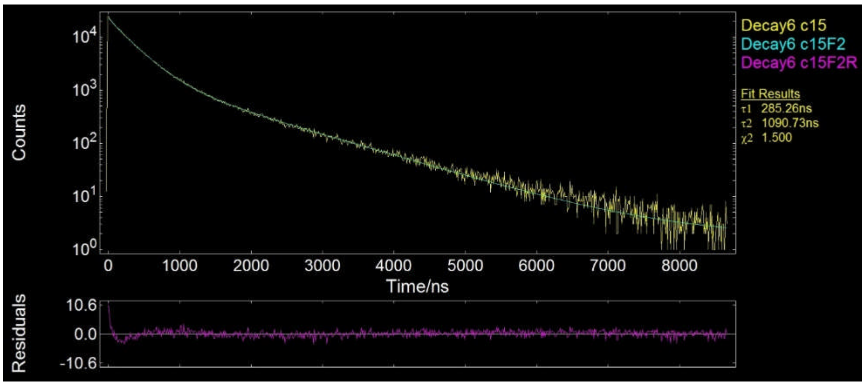

Figure S35. Lifetime decays of 1.4 after excitation at 379 nm in degassed MeCN at 298 K (λ_{em} = 596 nm).

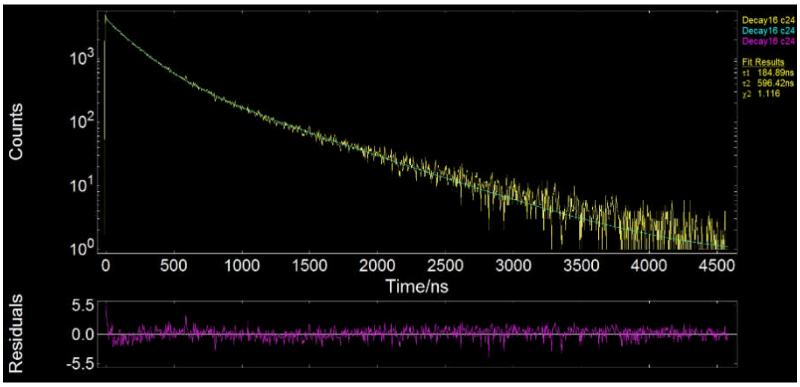

Figure S36. Lifetime decays of 2.3 after excitation at 379 nm in degassed MeCN at 298 K (λ_{em} = 608 nm).

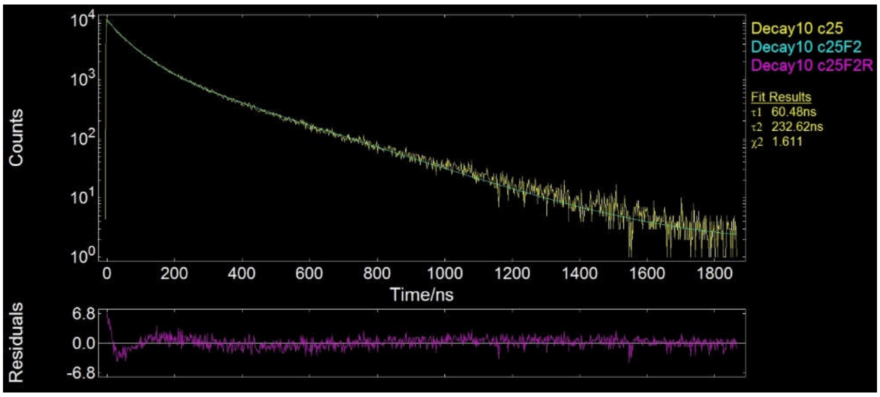

Figure S37. Lifetime decays of 2.4 after excitation at 379 nm in degassed MeCN at 298 K (λ_{em} = 611 nm).

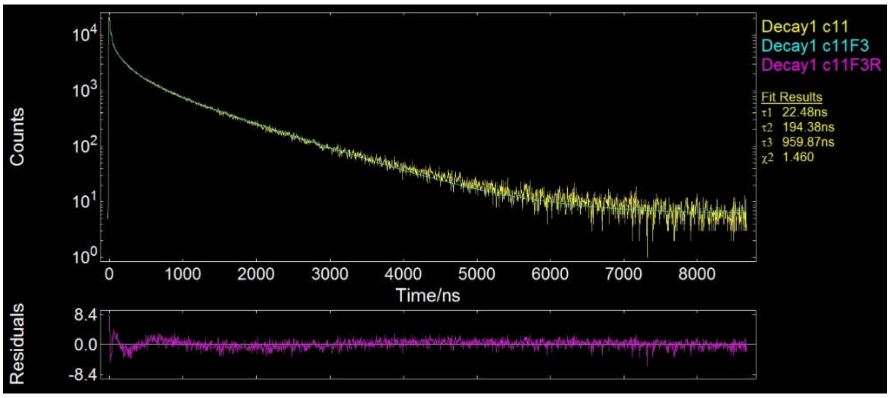

Figure S38. Lifetime decays of 1.1 after excitation at 379 nm in PMMA-doped film at 298 K (λ_{em} = 565 nm).

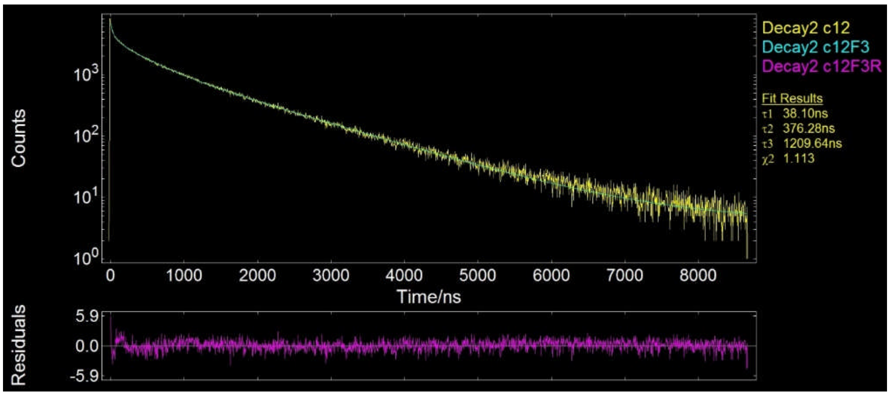

Figure S39. Lifetime decays of 1.2 after excitation at 379 nm in PMMA-doped film at 298 K (λ_{em} = 566 nm).

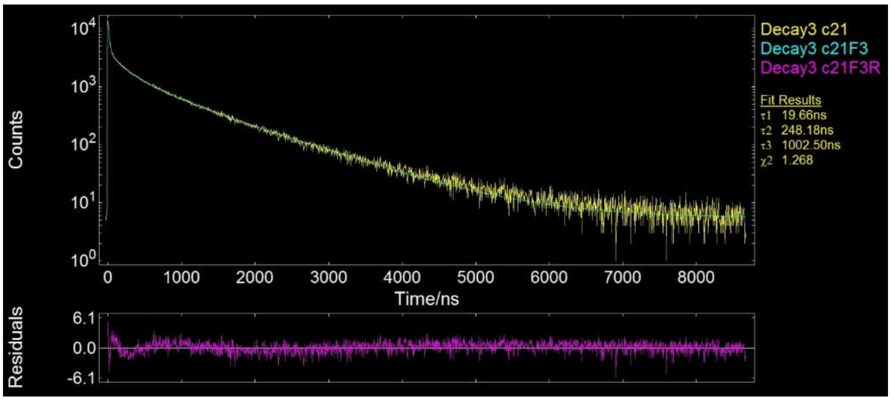

Figure S40. Lifetime decays of 2.1 after excitation at 379 nm in PMMA-doped film at 298 K (λ_{em} = 625 nm).

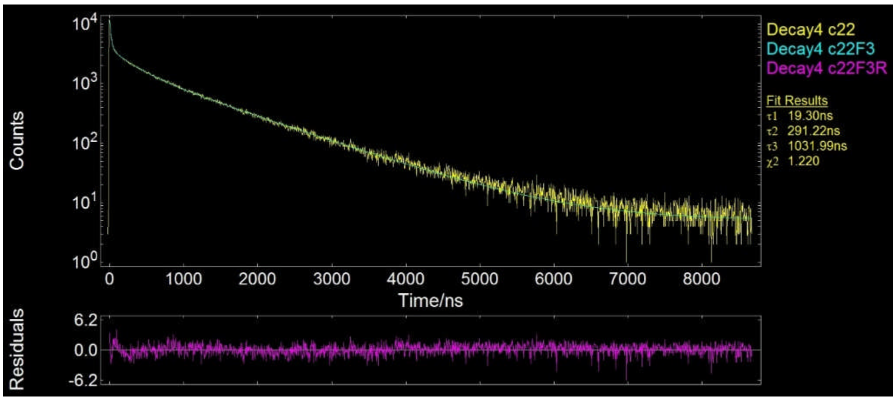

Figure S41. Lifetime decays of 2.2 after excitation at 379 nm in PMMA-doped film at 298 K (λ_{em} = 601 nm).

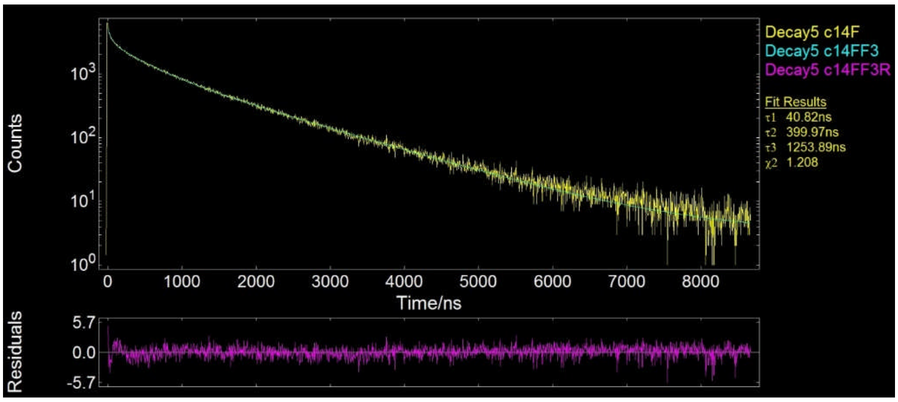

Figure S42. Lifetime decays of 1.3 after excitation at 379 nm in PMMA-doped film at 298 K (λ_{em} = 554 nm).

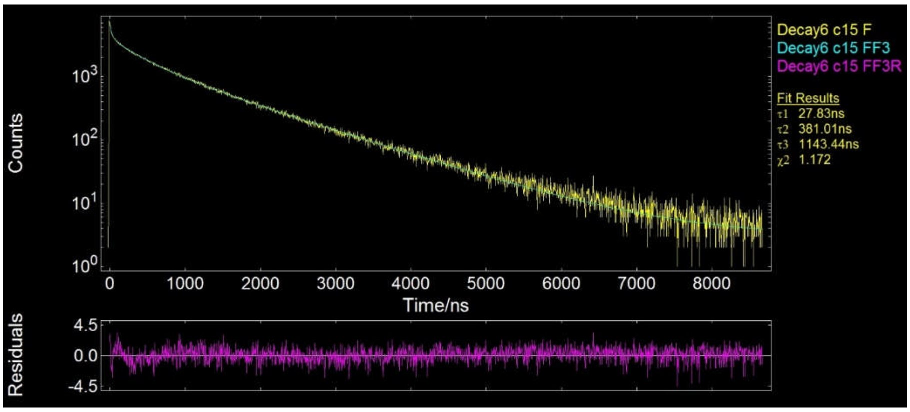

Figure S43. Lifetime decays of 1.4 after excitation at 379 nm in PMMA-doped film at 298 K (λ_{em} = 563 nm).

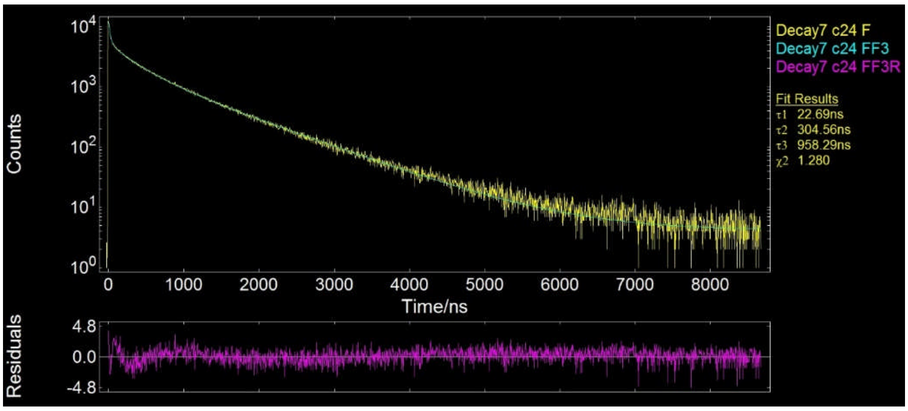

Figure S44. Lifetime decays of 2.3 after excitation at 379 nm in PMMA-doped film at 298 K (λ_{em} = 608 nm).

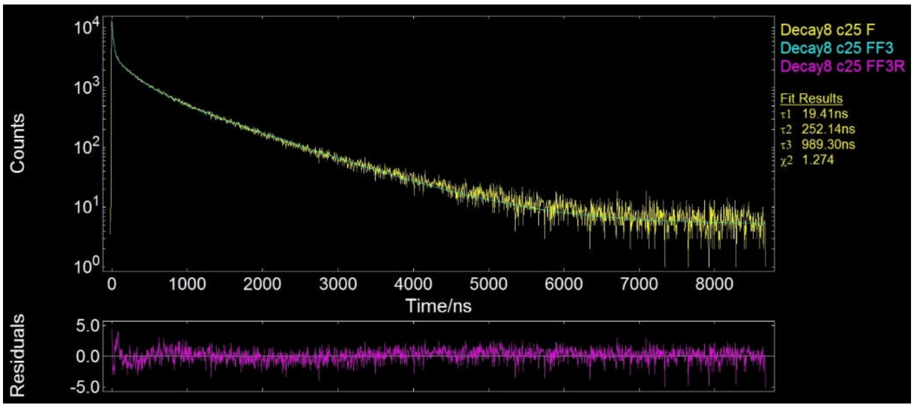

Figure S45. Lifetime decays of 2.4 after excitation at 379 nm in PMMA-doped film at 298 K (λ_{em} = 611 nm).

5. Electrochemical properties

Table **S2**. Electrochemical properties.

Complex	E_{ox}[c] / V	E_{ox}[c] / V	E_{red}[a,b] / V	E_{red}[a,b] / V
**1.1**	1.24	-	-1.54	-
**1.2**	1.26	-	-1.55	-
**2.1**	1.30	-	-1.06	-1.66
**2.2**	1.33	-	-1.15	-1.70
**1.3**	1.28	1.74	-1.42	-
**1.4**	1.29	1.73	-1.50	-
**2.3**	1.34	1.68	-1.06	-1.52
**2.4**	1.33	1.66	-1.09	-1.57
**L2**	-	-	-1.08	-

[a] CV traces recorded in MeCN solution with 0.1 M (*n*-$Bu_4N)PF_6$ at 298 K at 50 $mV{\cdot}s^{-1}$. Values are in V *vs.* SCE (Fc/Fc^+ *vs.* SCE = 0.38 V). [b] $E_{1/2} = (E_{pa} + E_{pc})/2$ and result from one-electron processes. [c] Irreversible or quasi-reversible oxidation peak potentials.

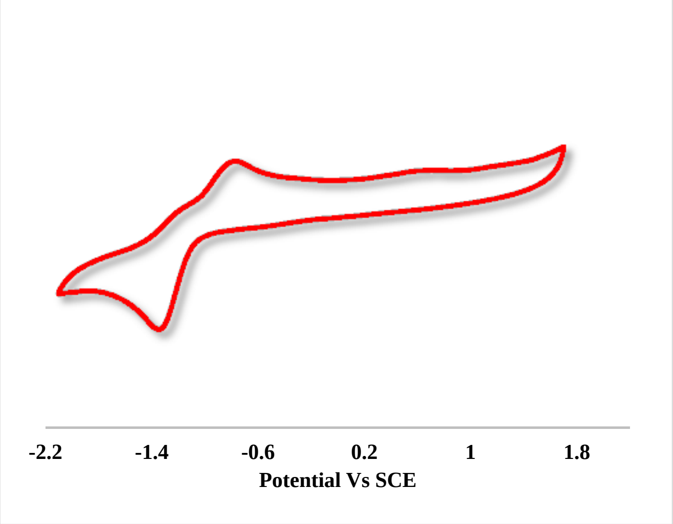

Figure S46. CV of L2 recorded at 298 K in degassed MeCN solution containing n-NBu$_4$PF$_6$ as the supporting electrolyte and using Fc/Fc$^+$ as an internal standard (Fc/Fc$^+$ = 0.38 V in MeCN with respect to SCE).[4]

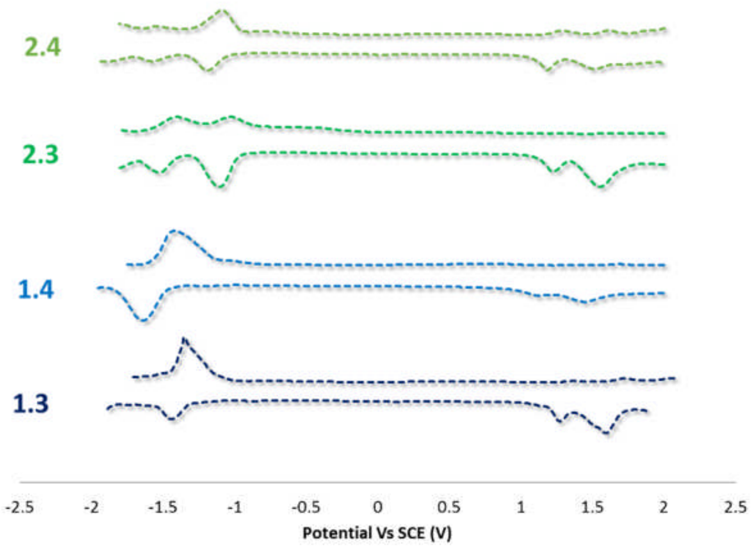

Figure S47. DPV of 1.3, blue line; 1.4, light-blue line; 2.3, green line and 2.4, light-green line. The spectra were recorded at 298 K in degassed MeCN solution containing n-NBu$_4$PF$_6$ as the supporting electrolyte and using Fc/Fc$^+$ as an internal standard (Fc/Fc$^+$ = 0.38 V in MeCN with respect to SCE).[4]

References

1. G. A. Crosby and J. N. Demas, *J. Phys. Chem.*, 1971, **75**, 991-1024.
2. W. H. Melhuish, *J. Phys. Chem.*, 1961, **65**, 229-235.
3. A. M. Brouwer, *Pure Appl. Chem.*, 2011, **83**, 2213-2228.
4. V. V. Pavlishchuk, A.W. Addison, *Inorg. Chim. Acta* 2000, **298**, 97.
